# Visual perception of rotated chromatic and achromatic 3D stimuli in goldfish (*Carassius auratus*)

**DOI:** 10.3758/s13420-025-00687-y

**Published:** 2025-11-12

**Authors:** Caroline M. DeLong, Kathryn Gardner, Jessica J. Wegman, Kenneth Tyler Wilcox

**Affiliations:** 1 Department of Psychology, College of Liberal Arts, Rochester Institute of Technology, 18 Lomb Memorial Dr., Rochester, NY 14623 USA; 2 Department of Psychology, College of Arts and Letters, University of Notre Dame, Notre Dame, IN 46556 USA; 3 Cornell Statistical Consulting Unit, Cornell University, Ithaca, NY 14853 USA

**Keywords:** Goldfish, Object constancy, Object discrimination, Visual perception

## Abstract

The ability to visually recognize objects despite differences in orientation would be advantageous for fish because they see objects from many viewpoints as they navigate their three-dimensional aquatic environment. We tested the ability of goldfish to recognize 3D chromatic and achromatic stimuli from four aspect angles in three rotation planes using a two-alternative forced-choice task. The fish were trained to discriminate between plastic models of a frog and turtle at 0°, then tested with the same objects at 0°, 90°, 180°, and 270°. In Experiments 1 and 2, the stimuli were presented in color, whereas in Experiment 3, the same stimuli were painted black. In Experiment 1, the fish performed significantly better than chance at all aspect angles (0°, ±90°, 180°) and in all three rotation planes. The goldfish displayed viewpoint-invariant performance in the picture plane, but showed enhanced performance at 0° for the two depth plane rotations, which suggests some viewpoint-dependent processes. In Experiment 2, performance accuracy was high regardless of whether the reinforced stimulus (S+) or the non-reinforced stimulus (S−) was rotated in the picture plane. In Experiment 3, two of four fish were successful in recognizing rotated achromatic stimuli. These results, taken together with other studies, suggest that goldfish more easily achieve visual object constancy when the stimuli contain surface features (color, texture, shading).

**Supplementary Information:**

The online version contains supplementary material available at 10.3758/s13420-025-00687-y.

Object constancy (i.e., shape or form constancy) refers to the ability to recognize 3D objects even when varying the viewpoint changes the 2D retinal image. This is considered a foundational visual task, and human infants as well as newborns of other species show evidence of recognizing objects across views (e.g., Bertenthal et al., 2013; Frick & Möhring, 2013; Wood & Wood, 2020). Fish, in their aquatic three-dimensional environment, have the opportunity to view objects from many different aspect angles (e.g., from the top, bottom, or side). It would be beneficial for fish to have a robust and flexible visual recognition system that is capable of identifying individuals, prey items, predators, and other objects despite changes in orientation.

Visual object constancy is indeed advantageous for species living in all kinds of environments and has been explored in a wide variety of animals. Mammals, including horses (Corgan et al., 2021; Hanggi, 2010), sheep (Kendrick et al., 1996), rodents (e.g., Minini & Jeffery, 2006; Sutherland, 1969), ferrets (Pollard et al., 1967), dogs (Siniscalchi et al., 2023), monkeys (e.g., Logothetis et al., 1994; Parr & Heintz, 2008), chimpanzees (e.g., Parr, 2011), sea lions (Mauck & Dehnhardt, 1997; Schusterman & Thomas, 1966), and dolphins (DeLong et al., 2020) have exhibited object constancy under many conditions. Birds, including chicks (e.g., Wood & Wood, 2020, 2024) and pigeons (e.g., Peissig et al., 2005; Spetch et al., 2001), have also shown evidence of object constancy with some limitations. Several species of fish, such as archerfish (Newport et al., 2018), cichlids (Schluessel et al., 2014), goldfish (e.g., DeLong et al., 2018; Mackintosh & Sutherland, 1963), and medaka (Wang & Takeuchi, 2017), show some evidence of object constancy. Even honeybees (Dyer & Vuong, 2008) have shown some level of success in recognizing objects visually from different viewpoints. In these studies, stimulus type (e.g., line drawings vs. shaded images, face vs. nonface objects) and methodological variables (e.g., number of trained views, which rotation plane is tested) were manipulated to explore the constraints on performance and the underlying mechanisms at work supporting this ability.

Testing object constancy typically involves presenting stimuli rotated along multiple axes (or rotation planes). A picture plane rotation, or a planar rotation, affords a view of the same object features for all views. For example, imagine you are viewing a picture of a person facing the camera. If you rotate that picture upside down, you still see the person’s face and the front of their body. However, if you rotate a view of a person in the depth plane around the *y*-axis or the *x*-axis, new features appear, such as the back of their head and body or the bottoms of their shoes. According to some studies with human participants, there are similar patterns of performance (accuracy decreases and reaction time slows as objects are rotated away from a trained view) across picture and depth plane rotations (Tarr, 1995; Tarr & Pinker, 1989). In studies where the same subjects have discriminated between the objects rotated in multiple planes, some organisms show greater accuracy (e.g., bottlenose dolphins in DeLong et al., 2020) or faster trial times (e.g., goldfish in Wegman et al., 2022) for picture plane rotations versus depth plane rotations.

Rotation scheme is another factor that has been occasionally explored in object constancy studies using two-alternative forced-choice tasks (Bowman & Sutherland, 1969; DeLong at el., 2018; Schusterman & Thomas, 1966). The two choices can both be rotated to the same aspect angle (“matched rotation scheme”), or only the positive stimulus (S+) or negative stimulus (S−) can be rotated (with the other stimulus presented at the training viewpoint). Most object constancy studies tend to use the matched rotation scheme. But manipulating rotation scheme could give insight into whether the organism is preferentially attending to the S+ or S−, as well as which stimulus features the organism is choosing to prioritize when making decisions. Schusterman and Thomas (1966) found that a single sea lion subject viewing black shapes on a white background performed somewhat worse when just the S+ was rotated (*M* = 69%) compared with when just the S− was rotated (*M* = 96%) or when both stimuli were rotated (*M* = 84%). When only the S− is rotated, the S+ is easy to recognize (it is at the trained orientation) so this might be considered the easiest situation with highest expected accuracy if the animal looks preferentially at the S+. Schusterman and Thomas (1966) concluded that rotation scheme made only a “slight” difference but did not present a statistical comparison of accuracy across the rotation schemes. Bowman and Sutherland (1969) found that the mean accuracy of goldfish when either the S+ or S− were rotated was 76% versus 80% for the matched rotation scheme, and one of the three groups of fish failed to discriminate between shapes when only one stimulus was rotated (results do not specify whether it was the S+ or S−). They concluded that the animals’ overall pattern of performance suggested that they were paying attention to the relative number of points in the top halves of the “W” and “V” stimuli. DeLong et al. (2018) found that goldfish viewing 2D stimuli performed significantly better under the matched rotation scheme (*M* = 66%) versus when only the S+ was rotated (*M* = 58%), but the results were inconclusive because the fish had failed to discriminate between the stimuli at the trained aspect for the “S+ only” rotation test.

Comparing both accuracy and trial times on different rotation schemes may also shed light on how organisms are accomplishing the object constancy task and how they are representing the stimuli. There may be an advantage to the matched rotation scheme if organisms are examining both the S+ and S− and seek to compare them at the same aspect angle. Longer trial times (or lower accuracy) when only one stimulus is rotated versus in the matched rotation scheme could suggest they are “mentally rotating” one stimulus into correspondence with the other stimulus—a process used by humans (Shepard & Metzler, 1971). A typical mental rotation task utilizes two stimuli in a same/different task, where a subject must decide whether two rotated shapes are identical or different (in a “different” trial, Stimulus 2 is usually a mirror image of Stimulus 1). Like the stimuli in the nonmatched rotation scheme in the current study, the two stimuli in a mental rotation task are rotated to different aspect angles. A process of mental rotation is invoked when the reaction time increases (and/or accuracy decreases) as a function of aspect angle. There is evidence for mental rotation in some nonhuman animals (e.g., rhesus monkeys in Hassett et al., 2022; sea lions in Mauck & Dehnhardt, 1997) but not others (e.g., lion-tailed macaques in Burmann et al., 2005; pigeons in Hollard & Delius, 1982).

An important variable to consider in object constancy studies that has implications for how an animal is performing the task is the color of the stimuli. If there are color cues available, where the objects being discriminated are different colors (e.g., a red lobster vs. a blue frog), the animal may utilize color instead of shape to succeed. If the objects being discriminated are the same color (e.g., a black lobster and a black frog), the animal must use other stimulus characteristics such as shape (size and surface features may also be available). The inability to recognize rotated objects that are the same color may indicate that the animal may not be representing the shape of the object and is not achieving object constancy. Differential performance on chromatic and achromatic stimuli with higher accuracy viewing chromatic stimuli may indicate a preference for using color even if shape is discriminable. The animal’s decision of which stimulus features to use when discriminating between objects in any given study may also be influenced by the stimulus dimensions (2D or 3D), their species-typical reliance on color vision, and their prior experience with object recognition tasks.

Broadly speaking, there are two models of visual object recognition that account for object constancy: structural description models and view-based models (Peissig & Tarr, 2007). Structural description models, such as recognition-by-components, posit that objects are specified by connection of their component parts (“geons”) and that performance is viewpoint-invariant (Biederman & Gerhardstein, 1993). View-based models assert that when observers learn to discriminate between objects at trained viewpoints, recognizing those same objects at novel viewpoints carries a cost in accuracy and time (e.g., Tarr, 1995; Tarr & Pinker, 1989). Further, these costs can be systematically related to the distance from the trained view (e.g., Tarr & Pinker, 1989). It is possible that object recognition is initially viewpoint-dependent, and becomes more viewpoint-invariant with greater experience. Another possibility is that viewpoint-invariant and viewpoint-dependent information is encoded concurrently (Tarr & Hayward, 2017). Some neurophysiological data supports the idea that there are more view-tuned neurons than view-invariant neurons, and pooling the responses of assemblies of neurons enables invariance in the primate brain (Logothetis et al., 1995). In contrast, there is evidence that chicks can build view-invariant object representations from a single view (Wood & Wood, 2020).

Early studies of object constancy conducted with goldfish utilized planar-rotated 2D black or white stimuli such as squares, rectangles, and letters (Bowman & Sutherland, 1969, 1970; Mackintosh & Sutherland, 1963; Sutherland & Bowman, 1969). In some cases, goldfish that were trained to discriminate between two stimuli could transfer that discrimination to rotated shapes (Bowman & Sutherland, 1969; Mackintosh & Sutherland, 1963), and in some cases they could not (Bowman & Sutherland, 1970; Sutherland & Bowman, 1969). Goldfish discriminated between planar-rotated 2D black geometric shapes (half circle vs. arrow) and black line drawings of animals (turtle vs. frog), with no overall difference in performance between the two stimulus types (DeLong et al., 2018). The fish succeeded at most novel aspect angles, but did not show strong performance overall (*M* = 67%; DeLong et al., 2018). These studies suggest that both viewpoint-dependent and viewpoint-invariant processes could be happening with goldfish.

Archerfish can discriminate 2D images of human faces regardless of whether they are presented in color or grayscale (Newport et al., 2016). When trained to recognize a frontal view of human faces (all the faces were skin tone color with dark eyes, facial contours, and hairlines), archerfish could recognize faces rotated by 30° or 60° but not all fish performed well at 90° (Newport et al., 2018). Because the different faces were not shown in different colors, presumably the archerfish relied on other features. Medaka fish trained to discriminate between 2D images of two conspecific fish or two 3D objects succeeded when the objects were rotated by 180° but not when the conspecific images were rotated by 180° (“face inversion effect”; Wang & Takeuchi, 2017). The pairs of 3D objects appear to have been the same color (both yellow or both green), which would eliminate color cues, but the conspecifics were shown to subjects via a prism system (individual fish vary color, patterning, and body shape) so color cues may have been available for those stimuli (Wang & Takeuchi, 2017).

Malawi cichlids were presented with 3D plastic animal models rotated in both the picture plane and depth planes (Schluessel et al., 2014). In Experiments 1 and 2, the subjects could discriminate between a lobster and a spotted fish (both brownish or both green/yellow tinted), and could discriminate between a reddish-brown lobster and a sparkly worm (at four aspect angles). This suggests fish may be able to recognize rotated 3D objects when the stimuli are largely the same color (but there were still slight variations in color and texture that fish could have used). In Experiments 3A and B, the cichlids learned to discriminate between a dark green turtle and frog, then continued to perform well when the stimuli were swapped for a light green pair. Similarly, performance was stable when switching from a yellow and orange turtle and frog to a gray pair. These results suggest that the fish were attending to shape differences instead of color, although the stimuli were not rotated. In Experiment 3C, the cichlids successfully discriminated between turtle and frog models that varied in color, shape, and texture at depth plane rotations (45°, 90°, 180°). Schluessel et al. (2014) suggested that the cichlids were attending primarily to shape and showed evidence of object constancy because the color of the turtles and the frogs varied. However, they never tested their subjects with stimuli that lacked all color cues and were exactly the same color (e.g., a black turtle and frog).

In the current study, our goal was to investigate the ability of fish to recognize rotated 3D chromatic and achromatic objects to see how well they perform an object constancy task without color cues. We presented goldfish with 3D plastic animal models (similar to the stimuli used by Schluessel et al., 2014) rotated in the picture plane and two depth planes, in three experiments utilizing a two-alternative forced-choice task. In Experiment 1, the fish were trained to discriminate between a green, black, and gray turtle and a yellow and red frog at 0° then tested with the same stimuli rotated at 0°, 90°, 180°, and 270° in three rotation planes. We used a matched rotation scheme during testing. We predicted that the fish would succeed at all aspect angles with chromatic stimuli (H1) given that other fish have largely succeeded at similar tasks with both 3D and 2D stimuli (Newport et al., 2016; Schluessel et al., 2014; Wang & Takeuchi, 2017).

To test the effect of prior exposure on performance, we gave a subset of the fish subjects continuous 24-hour exposure to the stimuli in their home tanks starting in pretraining and throughout Experiment 1. The stimuli, suspended from monofilament lines in the center of the tanks, could be explored from all aspect angles. If the fish are behaving consistently with viewpoint-dependent theories of object recognition, then the fish with prior exposure to the stimuli may perform better (i.e., higher accuracy or faster trial times) than the other fish because they would be able to experience more aspect angles and associate the different view angles with each other, building an object representation with multiple stored views (Tarr & Pinker, 1989). If the fish with prior exposure do not perform differently than the other fish and increased experience with different views did not confer a benefit, then it may be more consistent with the viewpoint-independent theory. For example, chicks can build view-invariant object representations from a single view (Wood & Wood, 2020). If prior exposure does not matter in this experiment, another possibility is that experience confers a benefit, but only when the stimuli are more difficult to discriminate.

In Experiment 2, we used a rotation scheme where either the reinforced stimulus (S+) or the nonreinforced stimulus (S−) was rotated during testing (nonmatched rotation scheme). We predicted that the fish would perform worse on Experiment 2 versus Experiment 1 (H2) because Bowman and Sutherland (1969) and DeLong et al. (2018) found that fish viewing 2D stimuli performed better under the matched rotation scheme. In Experiment 3, we painted the stimuli from Experiments 1 and 2 black to remove all color cues but retain the exact same size and shape differences. The fish were then tested with the stimuli rotated at 0°, 90°, 180°, and 270° in three rotation planes using the matched rotation scheme. We predicted that the fish would perform worse overall on Experiment 3 versus Experiment 1 because they had fewer cues to rely on by removing color (H3). We thought it possible that some fish may achieve success in Experiment 3 (e.g., at least some subjects could succeed at some aspect angles) because fish viewing rotated stimuli without color cues appeared to achieve object constancy under some conditions in past studies (Bowman & Sutherland, 1969; DeLong et al, 2018; Mackintosh & Sutherland, 1963; Newport et al., 2016).

## Experiment 1

### Method

#### Subjects

The subjects were eight goldfish (*Carassius auratus*), 4.5–12.5 cm in total length, obtained at local pet stores. Individual fish were identified by their coloration patterns (but sex was not determined phenotypically). Seven fish were approximately 4 months old at the start of the study and were experimentally naïve. One of these seven fish died during training. The eighth fish was approximately 5 years old at the start of the study, but failed to meet the training criteria in Training Stage 2 and did not advance to the test phase. Thus, six fish were included in the test phase.

Subjects were housed in pairs in 37.85 L tanks (50 cm in length × 26 cm in width × 31 cm in depth). The top of each tank contained one Aqueon® 10-watt mini-compact fluorescent light bulb on a 12-hour light/dark cycle. Each tank contained blue aquarium gravel and an Aqueon® Quiet Flow 10 filter unit that provided aerated, filtered, and conditioned water. All tanks were covered on three sides by blue cellophane on the exterior walls. Water temperature was kept between 20^◦^C–24^◦^C. Water changes (40%) were carried out on a weekly basis, and tanks were monitored regularly for pH, ammonia, nitrite, and nitrate levels with the API Freshwater Master Test Kit (MARS Fishcare Inc., Chalfont, PA). Additional tank changes were carried out occasionally for optimum water quality. Training and testing sessions were conducted during daylight hours (morning or afternoon), once per day, typically 5 days per week. On days with training or test sessions, to prevent overfeeding, subjects were fed only during the sessions. On weekdays when the fish were not trained or tested, they were fed with TetraFin flakes (Tetra GMBH, Melle, Germany) and API Premium pellets (Mars Fishcare Inc., Chalfont, PA).

#### Stimuli

**Fig. 1:**
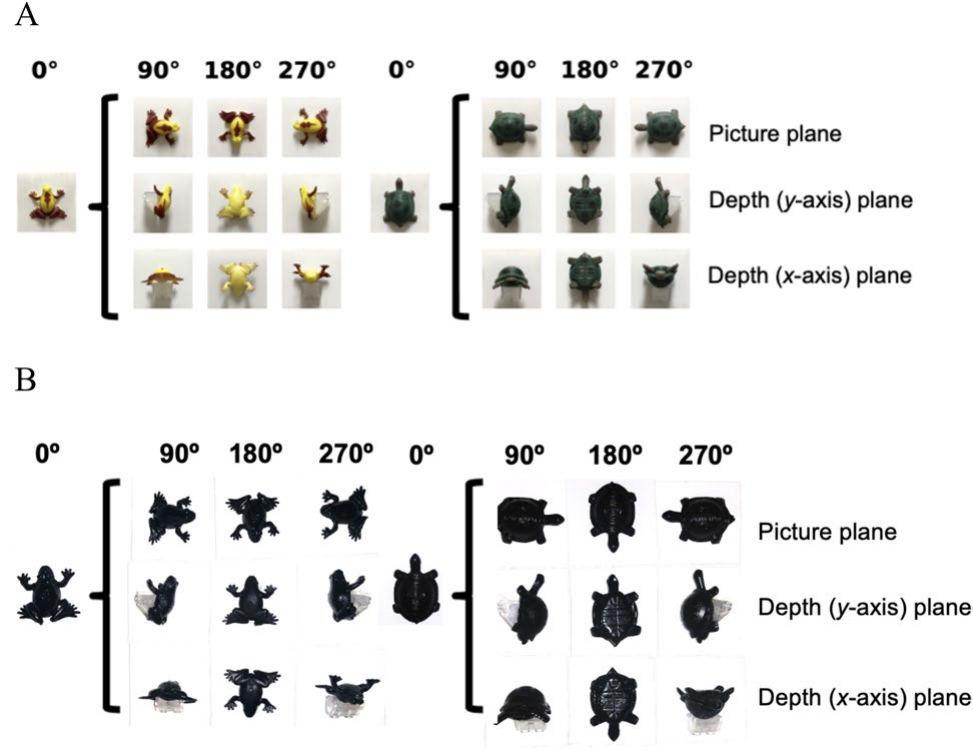
Stimuli used in all experiments. **A** All stimuli used in Experiments 1 and 2. **B** All stimuli used in Experiment 3. The dimensions of the frogs (2.8 cm in length × 3.4 cm in width × 1.2 cm in depth) and turtles (4.0 cm in length × 2.9 cm in width ×1.5 cm in depth) were the same in all experiments. In Experiments 1 and 3, the two choices were always shown at the same aspect angle (matched rotation scheme) whereas in Experiment 2 either the S+ or the S− was rotated while the other stimulus was shown at 0°

Figure 1A shows the 3D stimuli—plastic frogs (2.8 cm in length × 3.4 cm in width × 1.2 cm in depth) and turtles (4.0 cm in length × 2.9 cm in width ×1.5 cm in depth). The turtles were hollow, so they were lined with clear silicone waterproof sealant (Loctite, Rocky Hill, CT) to prevent bubbling when they were submerged in water (which would be an extraneous cue). There were slight differences between each stimulus due to production variation (e.g., each turtle had six spots on the shell; the spots had minor differences in shape and placement). Stimuli were purchased via Amazon.com (frogs: Oriental Trading, Omaha, NE; turtles: BT Group, London, UK).

All stimuli were mounted to the centers of pieces of white corrugated plastic (5.08 cm in length × 5.08 cm in width × 0.4 cm in depth) with Gorilla® super glue gel. The stimuli with aspect angles of 90° and 270° in both depth planes were attached to the corrugated plastic using a clear LEGO® piece (The Lego Group, Billund, Denmark) to secure the stimuli. The LEGO® pieces (1.0 cm in height × 1.5 cm in width × 1.5 cm in depth) were filled with the same clear silicone waterproof sealant used on the turtles to prevent bubbling when they were submerged in water. The corrugated plastic stimulus cards were attached to a white plastic corrugated stimulus board (25.5 cm in length × 16.5 cm in width × 0.4 cm in depth) with Velcro® hook and loop strips.

**Fig. 2:**
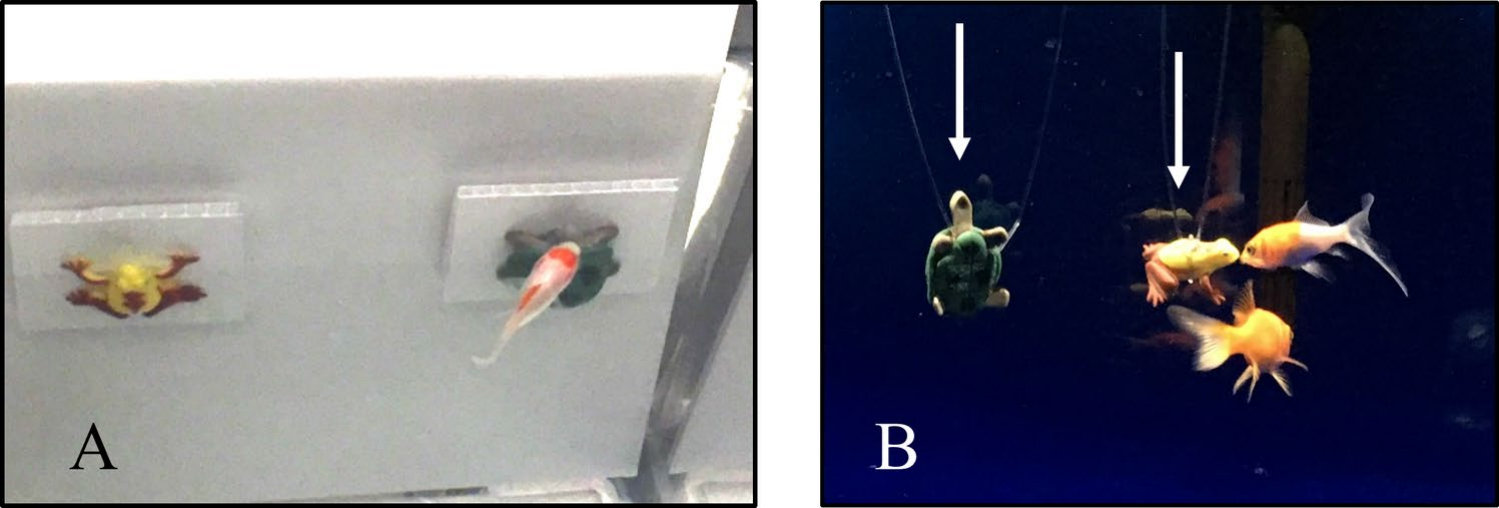
Fish choosing a stimulus during a test and exploring stimuli in their tank. **A** Photograph of fish during Training Stage 2 approaching its S+ in the test tank (both stimuli are presented at 0◦). The same setup is used for testing. **B** Two subjects were given long-term continuous exposure to the frog and turtle models in their home tank (arrows pointing at the models). The stimuli were hung on monofilament line approximately halfway between the gravel and the surface of the water, approximately 5 cm apart, and midway between the right and left tank walls

One unmounted frog and turtle were suspended by clear monofilament fishing line (Ande Inc., West Palm Beach, FL) in one home tank containing Fish 1 and Fish 6 (housed together). The frog and turtle were positioned 10 cm apart in the middle of the tank, about 18 cm deep (see Fig. 2B). These two stimuli were mounted in the home tank when Fish 1 and Fish 6 began pretraining and remained in the tank 24 hours per day throughout Experiments 1 and 2 so the fish would have continuous access to the stimuli and could explore them from all orientations.

#### Experimental setup

The same experimental setup was used as in Fig. 1 in Wegman et al. (2022). During training and testing sessions, subjects were moved to individual test tanks (14 L; 36.8 cm in length × 21.8 cm in width × 24.3 cm in depth) containing tap water treated with Aqueon® water conditioner but no gravel. Blue cellophane covered all four exterior side walls to block external stimuli during testing. There were six identical test tanks and fish were rotated among the six tanks. During training and testing sessions, the stimulus board was inserted in the front of the test tank and rested against the front interior tank wall. There were two different stimulus boards with the same dimensions. On one of the stimulus boards, a single stimulus could be mounted in the center of the board for Stage 1 training. On the other stimulus board, two stimuli could be presented side by side during Stage 2 training or the testing stage.

#### Procedure

Sessions were conducted once per day, around the same time of day, with typically five sessions per week. Test tanks were filled with water with the same approximate temperature as the home tanks and water conditioner, then the fish were transferred from the home tank to the test tank in a small bowl filled with water from the test tank. Fish acclimated to the test tank for a minimum of 15 min before testing began. Before the stimulus board was placed in the test tank, the fish was guided into the back center of the test tank by following the experimenter’s finger (the experimenter would drag a finger through the water to the back, gently tap on the water in the back of the tank or tap the back wall of the tank). Thus, the fish was swimming in the back of the tank when the trial began and encouraged the fish to examine both stimuli on the board before making a choice once it swam to the front of the tank.

The fish were reinforced for correct choices with food paste (a mixture of TetraFin flakes and water) similar to what has been used in other studies (DeLong at el., 2018; Siebeck et al., 2009; Wegman et al., 2022). Food paste was delivered to the fish using a 1.0 mL Luer-Lok tip syringe (BD, Franklin Lakes, NJ), and 0.01 mL of food paste was delivered for each correct choice. The fish had to tap (bite or push with its mouth) the rewarded stimulus (S+) once before the food reward was given. The experimenter, standing in the front-center of the tank, held the syringe out of sight from the fish behind the test tank wall until a correct choice was made. As soon as the subject tapped once on the rewarded stimulus, the experimenter lowered the syringe into the tank to the S+. Once the syringe was positioned near the S+, the fish ate the food paste. If a fish tapped the incorrect stimulus (S−) it was not reinforced and the board was withdrawn. The correction method was not used, so the fish was allowed to choose only one stimulus (S+ or S−) for each trial. The stimulus board was always removed from the test tank after the fish’s choice. During the intertrial interval of about 15–30 s. the fish’s choice (S+ or S−) and the trial time were recorded.

A single experimenter presented the stimulus board, delivered the food reinforcement, and recorded the data during training sessions. During test sessions, one experimenter presented the stimulus board and delivered the food reinforcement, while a second experimenter stood out of the view of the fish and timed the test trials using a stopwatch (ProCoach RS-013 Water Resistant Sports Stopwatch) by following “start” and “stop” verbal cues from the first experimenter. Five experimenters trained and tested the fish throughout the experiment.

##### Pretraining

After the fish acclimated to their home tanks for one to two weeks, they were introduced to the test tanks. They were placed in the test tanks and fed the same food they would get in the home tanks for two sessions on two consecutive days. Five days later, they began learning to eat from the syringe in the test tanks. All fish immediately ate from the syringe after five sessions (six trials per session).

##### **Training**

Four of the fish were reinforced for selecting the frog, and the other four fish were reinforced for selecting the turtle. During the training stages, the fish were only exposed to the stimuli at the 0° aspect angle. During Stage 1 training, the fish had to tap the S+ to receive a food reward when only the S+ but not the S− was presented. This stage lasted for 12 sessions with six trials per session. During Stage 2 training, the fish were presented with the S+ and S− in a two-alternative forced-choice task (Fig. 2A). The position of the S+ for each trial was determined using a modified pseudorandom Gellermann series in which the S+ was never shown more than two trials in a row on the same side to prevent potential side biases. The fish were given a food reward if they tapped their S+, and the board was removed with no punishment if they tapped their S−. The fish were prompted with the correct answer (putting the syringe in front of the correct stimulus before a choice to force the correct choice) if the fish was showing a side bias (persistently choosing either the left or right stimulus across several sessions). Prompted trials were not included in the reported choice accuracy for training trials. If a fish did not make a choice within 3 min in a training session, the board was removed and the trial was repeated. Training Stage 2 lasted for 30 sessions with six trials per session.

##### **Testing**

Test sessions included both the S+ and S− in a two-alternative forced-choice task like in Training Stage 2, but with added novel stimuli. There were 24 test sessions (six trials per session) in each of three test blocks for a total of 72 test sessions (432 trials). For the first two trials of every session, the 0° stimuli (used during training) were presented. For the last four trials, the 0°, 90°, 180°, and 270° aspect angles were presented in random order. In the test trials, both stimuli were rotated (e.g., both the S+ and S− would be rotated to 90° in a 90° trial). The S+ was on each side equally often within a test session (three times on the right, three times on the left), with no more than two appearances of S+ on the same side. As in Stage 2 training, the fish were given a food reward if they tapped their S+, and the board was removed with no punishment if they tapped their S−. There were three different test blocks, where all the trials within a test block contained stimuli from only one rotation plane. The first test block included stimuli rotated in the picture plane, the second test block included stimuli rotated in the depth plane about the *y*-axis, and the third test block included stimuli rotated in the depth plane about the *x*-axis (see Fig. 1A).

##### Interim training

In between each block of test sessions, each fish had to complete at least seven Stage 2 training sessions (six trials per session) to show they could still master the task and to potentially remove any side biases that may have formed during the previous test block. The fish needed a minimum mean accuracy of 75% during interim training before they could move on to the next test block. The fish completed 42–72 trials and accuracy ranged from 76% to 100% in each interim training phase for each fish. Average accuracy across all fish for each interim training phase was as follows: between Test Blocks 1 and 2 (*M* = 87.7%, *SE* = 1.8%) and between Test Blocks 2 and 3 (*M* = 92.5%, *SE* = 1.7%).

#### Data analyses

Statistical analyses were performed using R (Version 4.5.0; R Core Team, 2025) using a Type I error rate of α = .05. In the case of post hoc comparisons, *p* values were adjusted using Tukey’s (1949) WSD method for pairwise comparisons, or Scheffé’s (1959) method if other contrasts were tested. We modeled trial times with a generalized linear model using an inverse Gaussian distribution with an inverse link function (for a discussion of the inverse Gaussian and other generalized linear models for modeling psychological response time data, see, e.g., Lo & Andrews, 2015). We modeled performance (i.e., discriminative accuracy) as measured by a choice to the S+ (correct choice) or to the S− (incorrect choice) with a logistic regression model. Multilevel models (e.g., Raudenbush & Bryk, 2002) with random effects were considered, but the number of tested fish prohibited convergence during model estimation, so between-fish differences were instead accounted for using fixed effects (McNeish & Kelley, 2019). To reflect the hierarchical longitudinal sampling design (a measurement burst design; e.g., Rast et al., 2012) in which trials were conducted within sessions and sessions were nested within combinations of fish and rotation plane, we accounted for change across trials, change across sessions, and differential change across trials across sessions (an interaction). We report the Kullback–Leibler (Cameron & Windmeijer, 1997) pseudo-*R*^2^ measure, *R*^2^_KL_, as a measure of overall effect size for the full model and semipartial-*R*^2^_KL_ to measure the contribution of each interaction or main effect for both logistic and inverse Gaussian regression models.

### Results

#### Training

##### **Performance accuracy**

Of the 1,080 training trials, the fish were prompted on 42 trials and did not respond on one trial, so we analyzed the 1,037 unprompted trials (this analysis included the Training Stage 2 sessions that occurred prior to testing and not the interim training trials). Their overall accuracy was significantly better than chance (i.e., 50%) based on a one-sample proportion test, *M* = 86.6%, *SE* = 1.1%, *Z* = 23.54, *p* < .001, 95% CI [84.7%, 100.0%]. We then fit a model with all main or linear effects, two-way, and three-way interactions of change over trials, change over sessions, fish, and S+ location, *R*^2^_KL_ = 24.8%. Using Type III likelihood ratio tests, none of the three-way interactions were statistically significant, all *p* > .25, all *R*^2^_KL_ < 0.7%.

Next, we tested two-way interactions. To assess change over trials or sessions, we examined interactions involving change over sessions and change over trials. The interaction between fish and change over trials, χ^2^(5) = 9.37, *p* = .524, *R*^2^_KL_ = 0.5%, was not statistically significant. Change over trials varied significantly across sessions, χ^2^(1) = 5.31, *p* = .021, *R*^2^_KL_ = 0.7%. At the beginning of training, there was no significant change in performance across trials (slope on log-odds scale: *b* = −0.01, *SE* = 0.12, 95% CI [−0.24, 0.23]). By the tenth training session (of 30), the odds of a correct choice improved by an average of 18% from trial to trial (slope on log-odds scale: *b* = 0.18, *SE* = 0.07, 95% CI [0.04, 0.33]). At the end of training, the odds of a correct choice improved by an average of 76% from trial to trial (slope on log-odds scale: *b* = 0.56, *SE* = 0.17, 95% CI [0.23, 0.89]).

**Fig. 3:**
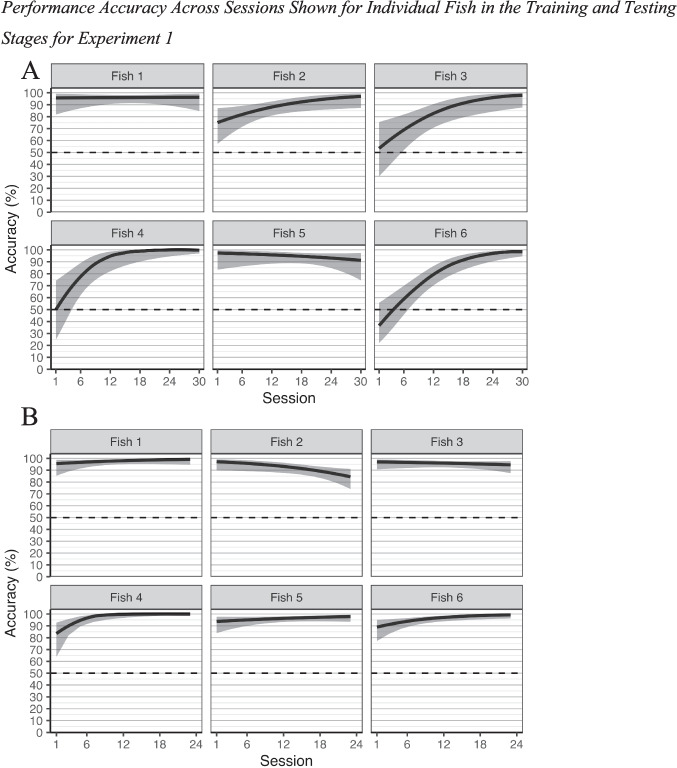
Performance accuracy across sessions shown for individual fish in the training and testing stages for Experiment 1**.** **A** Performance accuracy in the training stage before Experiment 1. **B** Performance accuracy in the testing stage of Experiment 1. Accuracies are shown along with 95% confidence bands

The interactions between change over sessions and S+ location, χ^2^(1) = 0.31, *p* = .577, *R*^2^_KL_ = 0.04%, and change over trials and S+ location, χ^2^(1) = 2.30, *p* = .129, *R*^2^_KL_ < 0.01%, were both nonsignificant. However, there was a significant interaction between change over sessions and fish, χ^2^(5) = 20.68, *p* = .001, *R*^2^_KL_ = 1.2%. As shown in Fig. 3, the odds of a correct choice for Fish 2 (slope on log-odds scale: *b* = 0.08, *SE* = 0.04, 95% CI [0.01, 0.15]) increased significantly by an average of 9% from session to session, corresponding to an average change from 74.8% (*SE* = 7.7%, 95% CI [57.1%, 86.9%]) performance at the start of training to 97.0% (*SE* = 2.3%, 95% CI [87.5%, 99.4%]) by the end of training. The odds of a correct choice for Fish 3 (slope on log-odds scale: *b* = 0.14, *SE* = 0.04, 95% CI [0.05, 0.23]) increased significantly by an average of 15% from session to session, corresponding to an average change from 52.0% (*SE* = 12.4%, 95% CI [29.1%, 74.1%]) performance at the start of training to 98.3% (*SE* = 1.6%, 95% CI [89.2%, 99.8%]) by the end training. The odds of a correct choice for Fish 4 (slope on log-odds scale: *b* = 0.27, *SE* = 0.08, 95% CI [0.11, 0.44]) increased significantly by an average of 31% from session to session, corresponding to an average change from 47.9% (*SE* = 13.4%, 95% CI [24.2%, 72.5%]) performance at the start of training to 100.0% (*SE* = 0.1%, 95% CI [97.6%, 100.0%]) by the end of training. The odds of a correct choice for Fish 6 (slope on log-odds scale: *b* = 0.17, *SE* = 0.04, 95% CI [0.10, 0.24]) increased significantly by an average of 19% from session to session, corresponding to an average change from 37.1% (*SE* = 8.8%, 95% CI [22.0%, 55.3%]) performance at the start of training to 98.9% (*SE* = 0.9%, 95% CI [94.9%, 99.8%]) by the end of training. The performance of Fish 1 and Fish 5 did not change significantly across sessions, both *p* > .40.

Finally, to check for potential side preferences, we tested for an interaction between fish and S+ position (left vs. right), This interaction was statistically significant, χ^2^(5) = 31.07, *p* < .001, *R*^2^_KL_ = 1.6%, but reflected a potential side preference for only one fish: By the end of training, Fish 3 performed significantly better when the S+ was on the right: 99.8% (*SE* = 0.4), CI [92.3%, 100.0%] versus when the S+ was on the left: 86.7% (*SE* = 6.1), CI [69.8%, 94.8%]. Fish 1, 2, 4, 5 and 6 did not exhibit a significant preference for either side, all *p* > .05. Importantly, all fish performed significantly better than chance at both S+ positions at the end of training, all *p* < .05. To test if prior exposure to the stimuli impacted performance, we compared the average performance of Fish 1 and Fish 6 (prior exposure; *M* = 93.1%) to Fish 2, 3, 4, and 5 (no prior exposure; *M* = 94.3%). There was no significant difference between performance (on the log-odds ratio scale) for the two sets of fish, log* OR* = −0.23, *SE* = 0.40, *Z* = −0.57, *p* = .570, 95% CI [−1.02, 0.56].

##### Trial time

In the training sessions, we modeled trial times (s) using an inverse Gaussian regression model with a canonical link function. Of the 1,037 unprompted training trials, three trials exceeded 60 s (one for Fish 3 in Session 4 and one for Fish 5 in Sessions 1 and 16) and were excluded from analysis, leaving 960 (92%) of the unprompted trials. The median trial time in the experiment was 3.2 s (median absolute deviation; *MAD* = 1.5 s). We then fit a model with main effects of fish and S+ location and their interaction, *R*^2^_KL_ = 22.1%; effects involving change over trials and sessions were inestimable and not included. Using Type III likelihood ratio tests, the interaction between fish and S+ location was statistically significant, χ^2^(5) = 14.78, *p* = .011, *R*^2^_KL_ = 2.1%. Only Fish 3 was significantly faster when the S+ was on the right (*M* = 2.4 s) compared with the left (*M* = 3.1 s), *Z* = 3.01, *p* = .003, whereas Fish 1, 2, 4, 5 and 6 did not exhibit a significant preference for either side, all *p* > .10.

To test if prior exposure to the stimuli impacted trial times, we compared the average trial time of Fish 1 and Fish 6 (prior exposure; *M* = 3.7 s when the S+ was on the left, *M* = 3.6 s when the S+ was on the right) with the other fish (no prior exposure; *M* = 3.5 s when the S+ was on the left, *M* = 3.3 s when the S+ was on the right) for each S+ position. There was no significant difference between trial times (on the inverse squared scale) for the two sets of fish when the S+ was on the left, *M* = 0.01 s^−2^, *SE* = 0.01, *Z* = 0.95, *p* = .344, 95% CI [−0.01, 0.02], or the right, *M* = 0.01 s^−2^, *SE* = 0.01, *Z* = 1.20, *p* = .229, 95% CI [−0.01, 0.03].

#### Test sessions

##### **Performance accuracy**

Of the 2,592 testing trials in Experiment 1, the fish completed 2,575 (99%). We analyzed the 2,575 completed trials. In the test sessions of Experiment 1, the overall accuracy was significantly better than chance (i.e., 50%) using a one-sample proportions test, *M* = 93.2%, *SE* = 0.5%, *Z* = 43.82, *p* < .001, 95% CI [92.3%, 100.0%]. We then fit a model with all main or linear effects, two-way, and three-way interactions of change over trials, change over sessions, fish, rotation plane, S+ orientation, and S+ location, *R*^2^_KL_ = 24.4%; three-way interactions among fish, rotation plane, and other variables and the three-way interaction among fish, change over sessions, and S+ orientation were inestimable and not included. Using Type III likelihood ratio tests, none of the three-way interactions were statistically significant, all *p* > .15, all *R*^2^_KL_ < 1.2%.

Next, we tested two-way interactions. To assess change over trials or sessions, we examined interactions involving change over sessions and change over trials. Neither the interaction between fish and change over trials, χ^2^(5) = 3.59, *p* = .610, *R*^2^_KL_ = 0.3%, and between rotation plane and change over trials, χ^2^(2) = 4.85, *p* = .088, *R*^2^_KL_ = 0.4%, were statistically significant. Change over trials did not vary significantly across sessions, χ^2^(1) = 0.24, *p* = .628, *R*^2^_KL_ = 0.02%, and change across trials was not statistically significant, χ^2^(1) = 3.81, *p* = .051, *R*^2^_KL_ = 0.3%. Because none of the effects involving change over trials were statistically significant, this suggested that performance did not vary across trials during testing. Interactions between change over sessions and S+ orientation, χ^2^(3) = 2.45, *p* = .485, *R*^2^_KL_ = 0.2%, and S+ location, χ^2^(1) = 0.00, *p* = .939, *R*^2^_KL_ = 0.0%, were both nonsignificant. However, there were significant interactions between change over sessions and fish, χ^2^(5) = 14.46, *p* = .013, *R*^2^_KL_ = 1.1%, and between change over sessions and rotation plane, χ^2^(2) = 6.93, *p* = .031, *R*^2^_KL_ = 0.5%.

Performance accuracy changed across sessions for three fish but did not change across sessions for the other three fish (see Fig. 3B). The odds of a correct choice for Fish 4 (slope on log-odds scale: *b* = 0.38, *SE* = 0.13, 95% CI [0.12, 0.64]) increased significantly by an average of 46% from session to session, corresponding to an average change from 87.4% (*SE* = 4.9%, 95% CI [74.5%, 94.3%]) performance at the start of a rotation plane test block to 100.0% (*SE* = 0.01%, 95% CI [99.2%, 100.0%]) by the end of the rotation plane test block. The odds of a correct choice for Fish 6 (slope on log-odds scale: *b* = 0.13, *SE* = 0.05, 95% CI [0.03, 0.23]) increased significantly by an average of 14% from session to session, corresponding to an average change from 90.1% (*SE* = 3.6%, 95% CI [80.4%, 95.3%]) performance at the start of a rotation plane test block to 99.3% (*SE* = 0.6%, 95% CI [96.2%, 99.9%]) by the end of the rotation plane test block. The odds of a correct choice for Fish 2 (slope on log-odds scale: *b* = −0.08, *SE* = 0.04, 95% CI [−0.16, −0.01]) decreased significantly by an average of 8% from session to session, corresponding to an average change from 96.9% (*SE* = 1.9%, 95% CI [89.8%, 99.1%]) performance at the start of a rotation plane test block to 84.4% (*SE* = 4.2%, 95% CI [74.2%, 91.1%]) by the end of the rotation plane test block. The performance of Fish 1, 3, and 5 did not change significantly across sessions, all *p* > .20.

The fish improved their performance across sessions for both depth rotation planes but not in the picture plane, where the fish were near 100% accuracy. Performance did not change significantly over sessions in the first rotation plane (picture plane, slope on log-odds scale: *b* = −0.03, *SE* = 0.04, 95% CI [−0.10, 0.04]). The odds of a correct choice increased significantly by an average of 14% from session to session in the second rotation plane (Depth (Y)), slope on log-odds scale: *b* = 0.13, *SE* = 0.04, 95% CI [0.06, 0.20]), corresponding to an average change from 88.6% (*SE* = 2.9%, 95% CI [81.5%, 93.2%]) performance at the start of the rotation plane to 99.2% (*SE* = 0.5%, 95% CI [97.3%, 99.8%]) by the end of the plane. The odds of a correct choice also increased significantly by an average of 18% from session to session in the third rotation plane (Depth (X)), slope on log-odds scale: *b* = 0.16, *SE* = 0.06, 95% CI [0.04, 0.28]), corresponding to an average change from 90.6% (*SE* = 2.6%, 95% CI [84.1%, 94.7%]) performance at the start of the rotation plane to 99.7% (*SE* = 0.4%, 95% CI [96.3%, 100.0%]) by the end of the plane. There was no significant difference between the rate of improvement over sessions between the Depth (Y) and Depth (X) rotation planes, *Z* = 0.41, *p* = .910.

We then tested for differences among fish and rotation planes. There was a significant interaction between fish and rotation plane, *χ*^2^(10) = 20.12, *p* = .028, *R*^2^_KL_ = 1.6%, as shown in Table S1. Fish 1, 4, 5, and 6 did not change their performance significantly across the three rotation planes, all *p* > .15. Fish 2 performed best in the first rotation plane (picture, *M* = 98.4%), performed significantly worse in the second rotation plane, Depth (Y), *M* = 81.5%, but may have improved (nonsignificantly) in the third rotation plane, Depth (X), *M* = 89.6%. Fish 3 performed best in the first rotation plane (picture, *M* = 98.8%) but had significantly lower performance in the second rotation plane, Depth (Y), *M* = 91.6%, and the third rotation plane, Depth (X), *M* = 93.2%. All fish performed significantly better than chance in each rotation plane, all *p* < .01. To test whether prior exposure to the stimuli impacted performance, we compared fish with prior exposure (Fish 1 and Fish 6) to fish with no prior exposure (Fish 2, 3, 4, and 5) in the picture rotation plane. There was no significant difference in performance based on prior exposure, *Difference in Log-Odds* = −0.63, *SE* = 0.54, *Z* = −1.18, *p* = .239, 95% CI [−1.68, 0.42].

Consistent with our first hypothesis (H1), all fish performed significantly better than chance in each S+ orientation, all *p* < .01, indicating that fish were able to discriminate successfully across aspect angles using a matched rotation scheme. Performance varied significantly among fish depending on the S+ orientation, χ^2^(15) = 30.37, *p* = .011, *R*^2^_KL_ = 2.4%, as shown in Table S2. Fish 1, 3, and 4 did not change their performance significantly across the four S+ orientations. Fish 2 performed significantly better at 0° (*M* = 98.4%) compared with 180° (*M* = 86.3%) and 270° (*M* = 84.2%) orientations. Fish 5 performed significantly better at 0° (*M* = 99.6%) compared with 90° (*M* = 88.6%) and 180° (*M* = 91.8%) orientations. Fish 6 performed significantly better at 0° (*M* = 99.4%) compared with 180° (*M* = 93.0%).

To examine the trajectories of change in performance as the S+ was rotated away from 0° for each fish whose performance varied over S+ orientations, we tested linear and quadratic trends in accuracy as the orientation rotated from 0° to ± 90° to 180° using Scheffé’s correction. Fish 2 demonstrated a significant linear decline in performance as the S+ was rotated away from 0°, *Z* = −3.45, *p* = .003, but no significant quadratic trend, *Z* = 2.11, *p* = .107. Fish 5 demonstrated a significant quadratic trend, *Z* = 2.70, *p* = .026, and associated linear trend, *Z* = −3.32, *p* = .004, as the S+ was rotated away from 0°. Fish 6 demonstrated a significant linear decline, *Z* = −3.36, *p* = .004, but no significant quadratic trend, *Z* = 1.26, *p* = .452, as the S+ was rotated away from 0°.

**Fig. 4:**
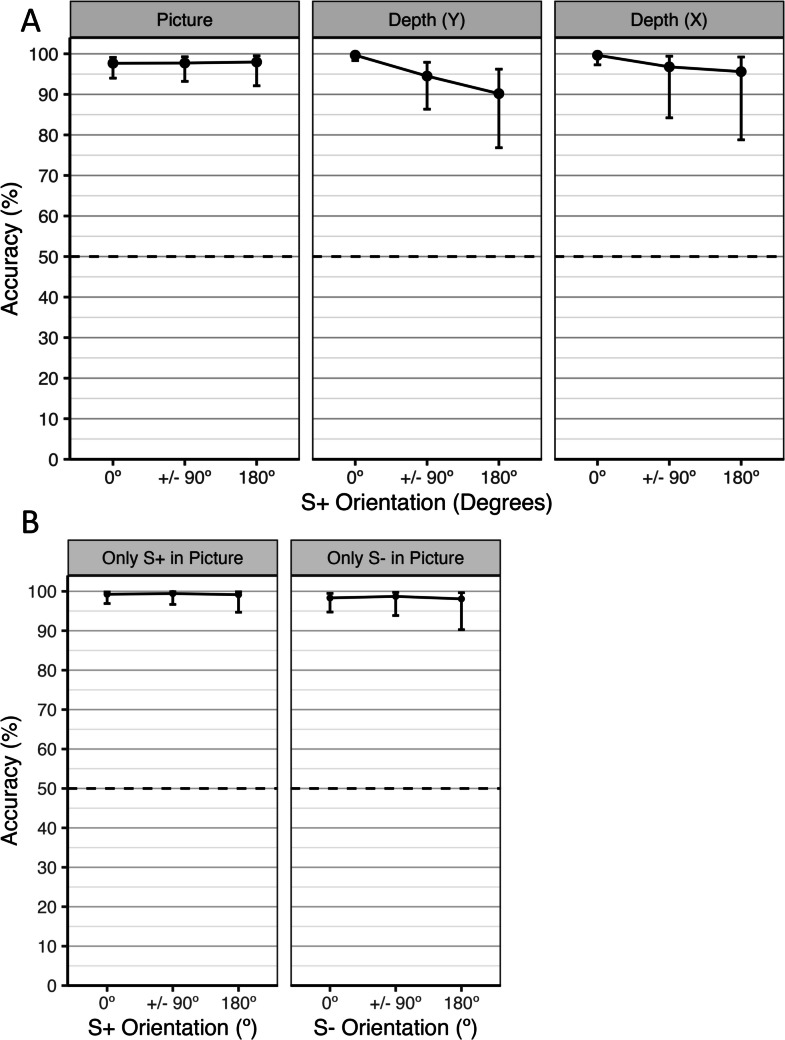
Performance accuracy as a function of rotation plane and aspect angle in Experiments 1 and 2. **A** Performance accuracy in the testing stage of Experiment 1. Accuracies are shown along with 95% Scheffé-adjusted simultaneous confidence intervals. Overlapping confidence intervals do not necessarily imply non-significant differences between orientations. **B** Performance accuracy in the testing stage of Experiment 2. Accuracies are shown along with 95% unadjusted simultaneous confidence intervals

Performance also varied across rotation planes depending on the S+ orientation, χ^2^(6) = 16.46, *p* = .012, *R*^2^_KL_ = 1.3%, as shown in Table S3 and Fig. 4. Performance did not vary across S+ orientations in the first rotation plane (picture, *M* = 97.9%). In the second rotation plane, Depth (Y), performance was significantly higher (*M* = 99.0%) at 0° than when the S+ was rotated away from 0° (*M* = 90.3%). Similar results were found in the third rotation plane, Depth (X), where performance was highest at 0° (*M* = 98.2%), but was significantly lower when the S+ was rotated away from 0° to 180° or 270° (*M* = 95.5%). To examine the trajectories of change in performance as the S+ was rotated away from 0° in each rotation plane, we tested linear and quadratic trends in accuracy as the orientation rotated from 0° to ± 90° to 180° in each plane using Scheffé’s correction. There was no evidence of linear or quadratic change in log-odds in the first rotation plane (picture), both *p* > .95. In the second rotation plane, Depth (Y), there was a significant quadratic trend in log-odds, *Z* = 2.75, *p* = .023, and an associated significant linear trend, *Z* = −5.63, *p* < .001. In the third rotation plane, Depth (X), there was a significant linear trend, *Z* = −4.22, *p* < .001, but no significant quadratic trend, *Z* = 2.41, *p* = .054.

Finally, to check for potential side preferences, we tested for an interaction between fish and S+ position (left vs. right), This interaction was statistically significant, χ^2^(5) = 26.50, *p* < .001, *R*^2^_KL_ = 2.1%, and is summarized in Table S4. Fish 1 and 5 performed better when the S+ was on the left, Fish 3 performed better when the S+ was on the right, and Fish 2, 4, and 6 did not exhibit a significant preference for either side. However, all fish performed significantly better than chance at both S+ positions, all *p* < .001, and the differences in performance between positions were less than 5% in magnitude except for Fish 3 (11.8%). The interaction between S+ orientation and S+ location was not significant, χ^2^(3) = 2.53, *p* = .469, *R*^2^_KL_ = 0.2%. Because of their involvement in significant interactions, we did not test main effects for fish, rotation plane, or S+ orientation.

##### **Trial time**

In the test sessions of Experiment 1, we modeled trial times (s) using an inverse Gaussian regression model with a canonical link function. Of the 2,575 completed trials, trial times (s) were available for 1,953 (76%) of the trials; we modeled these available trial times. The median trial time in the experiment was 3.5 s (median absolute deviation; *MAD* = 2.0 s). We fit a model with main effects of fish, rotation plane, S+ position, and S+ orientation and an interaction between fish and rotation plane, *R*^2^_KL_ = 24.9%; change over sessions and trials and higher-order interaction effects were inestimable and not included.

We first tested for differences among fish and rotation plane. There was a significant interaction between fish and rotation plane, χ^2^(10) = 24.62, *p* = .006, *R*^2^_KL_ = 1.9%, as shown in Table S1 and Figure S1. Trial times for Fish 3, 5, and 6 did not change significantly across the three rotation planes. Fish 1 was significantly faster in the first rotation plane (picture, *M* = 3.4 s) than in the third rotation plane, Depth (X), *M* = 5.0 s, *Z* = 3.89, *p* = .002, but their trial times in the second rotation plane, Depth (Y), *M* = 4.4 s, did not differ significantly from their trial times in the first, *Z* = −2.66, *p* = .070, or third rotation plane, *Z* = 1.30, *p* = .641. Similarly, Fish 2 was significantly faster in the first rotation plane (picture, *M* = 5.1 s) than in the third rotation plane, Depth (X), *M* = 7.6 s, *Z* = 3.56, *p* = .006, but their trial times in the second rotation plane, Depth (Y), *M* = 6.0 s, did not differ significantly from their trial times in the first, *Z* = −1.58, *p* = .474, or third rotation plane, *Z* = 2.12, *p* = .210. Fish 4 was significantly faster in the first rotation plane (picture, *M* = 3.2 s) than in both the second rotation plane, Depth (Y), *M* = 5.3 s, *Z* = 4.98, *p* < .001, and the third rotation plane, Depth (X), *M* = 4.9 s, *Z* = 4.49, *p* < .001, but their trial times in the second and third rotation planes did not differ significantly, *Z* = −0.70, *p* = .921. To test whether prior exposure to the stimuli impacted trial times, we compared fish with prior exposure (Fish 1 and Fish 6) with fish with no prior exposure (Fish 2, 3, 4, and 5) in the picture rotation plane. There was no significant difference in average trial time based on prior exposure, *Mean Difference* = −0.01 s^−2^, *SE* = 0.01, *Z* = −1.11, *p* = .268, 95% CI [−0.03, 0.01].

Trial times did not vary significantly among S+ orientations, χ^2^(3) = 5.93, *p* = .115, *R*^2^_KL_ = 0.4%, as shown in Table S2. Finally, trial times did not vary significantly among S+ positions, χ^2^(3) = 5.93, *p* = .115, *R*^2^_KL_ = 0.4%.

### Discussion

In Experiment 1, goldfish achieved object constancy when viewing frog and turtle stimuli that varied in color. All of the fish performed better than chance at all aspect angles, and overall performance in the test phase was high (*M* = 93.2%). The fish also acquired this object discrimination swiftly. One-third of the fish subjects reached ceiling performance at the very beginning of the training phase, and the other subjects learned the task quickly (reaching ceiling performance within about 100 trials). During the test phase, five of the six fish maintained very high performance across test sessions (Fig. 3). Their acquisition profile and performance across the experiment suggests that this was a relatively easy task for the goldfish.

The performance of the goldfish as a function of aspect angle yields insight into how they represented the stimuli. Three of the fish did not have significant differences in performance as a function of aspect angle, which would suggest they formed viewpoint-independent representations of the stimuli. However, the other three fish performed significantly better at 0° (training aspect) compared with other aspects (90°/180°/270°), although they performed better than chance at all angles. This pattern of performance is suggestive of view-based learning, and converges with a study showing chicks have enhanced recognition of familiar views when exposed to multiple views of an object (Wood & Wood, 2020). A likely explanation is that the fish in the current study had a mix of viewpoint-invariant and viewpoint-dependent representations. A bottlenose dolphin viewing rotated 2D stimuli (DeLong et al., 2020) and goldfish viewing rotated black and white 2D stimuli (DeLong et al., 2018) also showed a pattern of performance that may indicate both viewpoint-invariant and viewpoint-dependent processes.

The result showing the rotation plane of the stimuli had a significant effect on both time and accuracy in Experiment 1 can also shed light on how the fish represented the objects. The performance of the fish overall did not change over time during the picture plane test block, but it improved over time across both depth plane test blocks. Two of the six fish performed best in the picture plane test block compared with one or both of the depth plane test blocks, and three of the six fish had faster trial times in the picture plane test block compared with one or both of the depth plane test blocks. In addition, performance varied as a function of aspect angle only for the two depth planes (performance highest at 0°). Other studies have shown animals show faster trial times (Wegman et al., 2022) or greater accuracy (DeLong et al., 2020) on picture plane versus depth plane rotations. With a picture plane rotation, all features of the object present in the object during the training phase remain present at all novel aspect angles, whereas the same cannot be said for depth plane rotations (see Fig. 1). The size, shape, and available colors change with depth plane rotations. If an individual is using a diagnostic feature to discriminate between the stimuli (e.g., the pattern on the back of the frog or the shell of the turtle), and that feature becomes unavailable at a depth plane rotation (e.g., 90° Depth X), then that individual may take longer or be less accurate. That pattern of results may indicate some viewpoint-dependent processes at work. However, it is important to note that all the fish performed better than chance at all aspect angles in both depth plane rotation tests, so this again suggests a mix of viewpoint-dependent and viewpoint-independent processes.

In the test phase of Experiment 1, both stimuli were rotated to the same aspect angle (i.e., the “matched rotation scheme”). To test whether rotation scheme had an impact on performance or trial time, in Experiment 2 we presented the fish with the nonmatched rotation scheme in which only the S+ or S− were rotated (the other stimulus was presented at 0°). In Experiment 2, goldfish continued to view the same 3D stimuli that varied in color as in Experiment 1. Past studies have shown the fish seem to perform better using the matched rotation scheme (Bowman & Sutherland, 1969; DeLong et al., 2018), so we predicted lower performance in Experiment 2. Comparing trial time differences between the rotation schemes could give insight into whether fish might be engaging in a mental rotation process (Shepard & Metzler, 1971).

## Experiment 2

### Method

#### Subjects

Five of the same six fish subjects (Fish 1, 3, 4, 5, 6) were tested as in Experiment 1 (Fish 2 died before the start of Experiment 2). The fish were tested immediately (one day) after the completion of Experiment 1. All aquarium conditions were the same as in Experiment 1.

#### Stimuli

The stimuli were the same turtles and frogs rotated in the picture plane shown in Fig. 1A.

#### Experimental setup

The experimental setup was the same as in Experiment 1.

#### Procedure

Test sessions were conducted the same as in Experiment 1. There were two test blocks in Experiment 2. Each test block was composed of 24 sessions (six trials per session) as in Experiment 1. The first two trials of every session the 0° stimuli were presented, and the last four trials contained one trial each of 0°, 90°, 180°, and 270° presented in random order. In the first test block, only the S+ was rotated (90°, 180°, or 270°) and the S− was always shown at 0° (except in 0° trials where both S+ and S− were at 0°). In the second test block, only the S− was rotated (90°, 180°, or 270°) and the S+ was always shown at 0° (except in 0° trials where both S+ and S− were at 0°).

One day after the completion of the third test block in Experiment 1 (stimuli rotated in the depth plane around the *x-*axis), the fish completed seven Stage 2 training sessions (six trials per session) where all stimuli were shown at 0°. Then, fish completed the first test block of Experiment 2, followed by seven more training sessions, followed by the second test block of Experiment 2. Individual performance accuracy for each fish in these training sessions ranged from 90% to 100%. Average accuracy across all fish for each interim training plane was as follows: between the last test block in Experiment 1 and the first test block in Experiment 2 (*M* = 96.0%, *SE* = 1.4%), and between the two blocks in Experiment 2 (*M* = 99.5%, *SE* = 0.5%).

#### Data analyses

The data analyses were the same as in Experiment 1.

### Results

#### Performance accuracy

Of the 1,440 testing trials in Experiment 2, the fish completed 1,439 (99.9%). We analyzed the 1,439 completed trials. In the test sessions of Experiment 2, the overall accuracy was significantly better than chance (i.e., 50%) using a one-sample proportions test, *M* = 97.6%, *SE* = 0.4%, *Z* = 36.09, *p* < .001, 95% CI [96.8%, 100.0%]. We compared performance between Experiment 1 and Experiment 2 with a fixed-effects meta-analysis of the correct and incorrect choices in both experiments using the log-odds ratio (e.g., Fleiss & Berlin, 2009). Counter to our hypothesis (H2) that performance would be worse overall in a nonmatched rotation scheme compared with a matched rotation scheme, performance was significantly better in Experiment 2 (*Accuracy* = 97.6%) than in Experiment 1 (accuracy = 93.2%), log-odds ratio = 1.10, *SE* = 0.19,* Z* = 5.76, *p* < .001, 95% CI [0.72, 1.47].

As in Experiment 1, we then fit a model with all main or linear effects, two-way, and three-way interactions of change over trials, change over sessions, fish, test block (Block 1: only the S+; Block 2: only the S− was rotated), S+ orientation, and S+ location, *R*^2^_KL_ = 15.7%; two-way and three-way interactions involving fish and test block, fish and S+ position, fish and S+ orientation, test block and S+ orientation, S+ position and S+ orientation, and change over trials and fish could not be estimated and were not included. Using Type III likelihood ratio tests, the three-way interaction between test block and change over sessions and change over trials was not statistically significant, *χ*^2^(1) = 0.06, *p* = .806, *R*^2^_KL_ = 0.02%.

Next, we tested two-way interactions. To assess change over trials or sessions, we examined interactions involving change over sessions and change over trials. As in Experiment 1, interactions between test block and change over trials, χ^2^(1) = 1.27, *p* = .432, *R*^2^_KL_ = 0.4%, change over trials and sessions, χ^2^(1) = 0.11, *p* = .741, *R*^2^_KL_ = 0.03%, were not statistically significant. There was also no statistically significant change across trials, χ^2^(1) = 0.00, *p* = .993, *R*^2^_KL_ = 0.00%. Because none of the effects involving change over trials were statistically significant, this suggested that performance did not vary across trials during testing. The interactions between change over sessions and S+ orientation was not significant, χ^2^(3) = 2.75, *p* = .432, *R*^2^_KL_ = 0.8%.

Unlike Experiment 1, the interaction between change over sessions and fish, χ^2^(4) = 2.16, *p* = .707, *R*^2^_KL_ = 0.7% was not statistically significant and there were no significant differences in performance among fish, χ^2^(4) = 5.81, *p* = .214, *R*^2^_KL_ = 1.8%, although all fish performed significantly better than chance in each test block (Table S1). There were no significant differences in performance among S+ orientations, χ^2^(3) = 2.56, *p* = .464, *R*^2^_KL_ = 0.8%, and performance was significantly better than chance for each S+ orientation (see Fig. 4B).

#### Trial time

In the test sessions of Experiment 2, we modeled trial times (s) using an inverse Gaussian regression model with a canonical link function. Of the 1,439 completed trials, trial times were available for 1,050 (73%) of the trials; we modeled these available trial times. The median trial time in the experiment was 3.6 s (median absolute deviation; MAD = 1.8 s). We compared the log-ratio of the average trial times between Experiment 1 and Experiment 2 with a fixed-effects meta-analysis (Hedges et al., 1999; Lajeunesse, 2011). The typical trial time was significantly slower (6%) in Experiment 2 than in Experiment 1, ratio = 1.06, *SE* = 0.02,* Z* = 3.47, *p* < .001, 95% CI [1.03, 1.10]. We fit a model with main effects of fish, test block, S+ position, and S+ orientation, an interaction between fish and test block, and—unlike in Experiment 1—interactions between test block and S+ orientation, test block and S+ position, and fish and S+ orientation, *R*^2^_KL_ = 36.1%; change over sessions and trials and other second- or higher-order interaction effects were inestimable and not included.

We first tested for differences among fish and test block. There was a significant interaction between fish and test block, χ^2^(4) = 44.57, *p* < .001, *R*^2^_KL_ = 4.6%, as shown in Table S1. Trial times for Fish 1, 5, and 6 did not change significantly as a function of test block. Fish 3 was significantly faster in Block 2 when only the S− was rotated (*M* = 2.4 s) than in Block 1 when only the S+ was rotated (*M* = 2.8 s), *Z* = −2.11, *p* = .035. Conversely, Fish 4 was significantly faster when in Block 1 when only the S+ was rotated (*M* = 4.0 s) than in Block 2 when only the S− was rotated (*M* = 6.9 s), *Z* = −5.79, *p* < .001. The interaction among fish and S+ orientation was not statistically significant, χ^2^(12) = 15.82, *p* = .200, *R*^2^_KL_ = 1.6%.

Although there was a significant interaction between test block and S+ orientation, χ^2^(3) = 12.82, *p* = .007, *R*^2^_KL_ = 1.2%, none of the pairwise comparisons of trial times between S+ orientations were statistically significant using Scheffé’s correction, all *p* > .20. We also tested linear and quadratic trends in trial time as the orientation rotated from 0° to ± 90° to 180° in each plane using Scheffé’s correction, neither of which was statistically significant in either plane, all *p* > 0.15. The interaction between test block and S+ position was not statistically significant, χ^2^(1) = 0.29, *p* = .592, *R*^2^_KL_ = 0.03%, but fish were significantly slower when the S+ was on the left (*M* = 3.8 s, *SE* = 0.1, 95% CI [3.7, 4.0]) than on the right (*M* = 4.1 s, *SE* = 0.1, 95% CI [4.0, 4.4]), *Z* = −3.07, *p* = .002.

### Discussion

In Experiment 2, the goldfish again displayed object constancy and performed at near ceiling level (*M* = 97.6%) while viewing the chromatic turtles and frogs in the nonmatched rotation scheme (either the S+ or S− was rotated while the other stimulus was presented at 0°). All fish showed viewpoint-independent performance—there was no difference in accuracy as a function of aspect angle. Contrary to our prediction, the fish performed significantly better overall in Experiment 2 than in Experiment 1. However, it is important to note that Experiment 2 included only the picture plane rotation. A more fair comparison would be to contrast the Experiment 1 picture plane test (*M* = 97.9%) with Experiment 2 (picture plane only, *M* = 97.6%; see Fig. 4). Viewed this way, there was no difference in performance as a function of rotation scheme.

One potential reason we found no deficit in performance with the nonmatched rotation scheme is because the fish were presented with the stimuli using the matched rotation scheme first, and had plenty of practice (more than 400 trials) viewing the rotated chromatic stimuli throughout Experiment 1. Also, their  performance did not increase in Experiment 2 because they were already near ceiling level in Experiment 1. Perhaps rotation scheme is irrelevant when the discrimination (e.g., between two rotated stimuli with color cues) is easy for the fish, and only becomes a relevant variable when the discrimination is more difficult. It is unfortunate we were unable to test the fish on the depth plane rotations in Experiment 2 (the fish were in other studies), but maybe future studies could continue to explore rotation schemes with more challenging stimuli and in different conditions (e.g., depth plane rotations).

Although there was no difference in accuracy as a function of rotation scheme, we found that trial times were overall slower in Experiment 2 than Experiment 1. One potential explanation of this is that the fish might have been taking extra time to mentally rotate one stimulus (e.g., at 90°) into correspondence with another stimulus (at 0°) before making their decisions. However, the goldfish in the current study did not show a pattern of performance that matched the classic mental rotation result in other ways – their trial times did not vary as a function of aspect angle in either Experiments 1 or 2. That their trial times were overall slower in Experiment 2 versus Experiment 1 might have been due to another factor (e.g., the novelty of viewing the stimuli in the nonmatched rotation scheme). Overall, there is not enough evidence to support a mental rotation process in this study. DeLong et al. (2018) tested goldfish with planar-rotated 2D stimuli and did not find a pattern of performance supporting mental rotation either.

In Experiment 3, to test whether the goldfish can achieve object constancy in the absence of color cues, we presented the fish with the same 3D stimuli from Experiment 1 painted black. Unlike the stimuli in Schluessel et al.’s (2014) study, these turtles and frogs were exactly the same color. Because there was effectively no difference in performance as a function of rotation scheme in Experiments 1 and 2, we used the matched rotation scheme in Experiment 3.

## Experiment 3

### Method

#### Subjects

The subjects were six goldfish (*Carassius auratus*), 5.0–12.0 cm in length, obtained in local pet stores. Subjects ranged in age from approximately 1 month–3 years old at the beginning of the study. Individual fish were identified by their color patterns, though sex was not determined. Subjects were housed and cared for as described in Experiment 1. Two fish had participated in Experiments 1 and 2 (Fish 4 and 5 from Experiment 1) and the other four fish had not. The time that had elapsed between the end of Experiment 2 and the start of Experiment 3 for these two fish was 33 months. All subjects participated in other experiments before the start of Experiment 3. Three of the six fish participated in an object constancy study using a two-alternative forced-choice task (but different black 3D stimuli) that lasted 5–8 months and was completed a year before Experiment 3 (C. DeLong, unpublished data), and all six fish participated in a study on long-term memory that lasted 4–6 months and was completed 6 months before Experiment 3 (C. DeLong, unpublished data).

#### Stimuli

The stimuli are shown in Fig. 1B. The stimuli were the exact same plastic models of turtles and frogs used in Experiments 1 and 2, but painted black with waterproof paint (Plasti Dip standard color black aerosol spray waterproof rubberized coating). Stimuli were mounted on the same white corrugated plastic stimulus cards using the same methods that were used as in Experiments 1 and 2. The stimulus cards were mounted with Velcro® hook and loop fasteners to a stimulus board made from white acrylic material (25.5 cm high ×16.5 cm wide × 0.4 cm thick).

#### Experimental setup

The setup was the same as in Experiments 1 and 2.

#### Procedure

The procedure was the same as in Experiments 1 and 2. Four experimenters participated in the training phase, and two participated in the test phase.

**Training.** Three of the fish were reinforced for selecting the turtle and the other three fish were reinforced for selecting the frog. The two fish that had participated in Experiments 1 and 2 were reinforced for selecting the same stimulus as in those experiments (the turtle for one, the frog for the other). Three fish did not need any pretraining and went straight to Training Stage 1.

The three fish who had not been trained to do a two-alternative forced-choice task were pretrained as described in Experiment 1.

Stage 1 training was conducted as in Experiment 1, except training sessions were interrupted for 6 months by the COVID-19 pandemic, so the fish completed 19–20 sessions after resuming training. Stage 2 training was conducted as in Experiment 1, except we extended the number of possible training sessions (from 50 to 61) to see if any fish could achieve the training criteria with extended practice. Fish had to meet two criteria to move onto the test: performance significantly above chance (*p* < .05 as determined by binomial tests) over all sessions and on the last seven sessions. Only four of the six fish met the training criterion to move onto the test, and their average performance accuracy on the last seven sessions was 67%.

**Testing.** Test sessions were conducted as in Experiment 1. As in Experiment 1, each test block contained 24 sessions, and all the trials within a test block contained stimuli from only one rotation plane. Only two test blocks were completed: Block 1 (stimuli rotated in the picture plane) and Block 2 (stimuli rotated in the depth plane around the *y*-axis). As in Experiment 1, interim training was conducted between test blocks. The fish achieved the training criterion in the interim training block between Test Block 1 and 2, but they failed to meet criterion after Test Block 2, so they did not move on to test Block 3 (showing pervasive side biases).

#### Data analyses

Statistical analyses were performed using R (Version 4.5.0; R Core Team, 2025) using a Type I error rate of α = .05. We modeled performance (i.e., discriminative accuracy) as measured by a choice to the S+ (correct choice) or to the S− (incorrect choice) with a logistic regression model. To account for temporal autocorrelation across repeated trials, we considered fitting multilevel models (e.g., Raudenbush & Bryk, 2002), but the small number of tested fish prohibited convergence during model estimation. Instead, we modeled between-fish differences using fixed effects (McNeish & Kelley, 2019) and accounted for autocorrelation over trials using heteroscedasticity-and-autocorrelation-consistent (HAC) robust variance-covariance estimation (Andrews, 1991) to avoid pseudoreplication. Results were robust to the use of HAC or standard estimation and conclusions remained unchanged. To reflect the hierarchical longitudinal sampling design in which trials were conducted within sessions and sessions were nested within combinations of fish and rotation plane, we accounted for change over time by modeling linear change over trials, sessions, and their interaction (e.g., Rast et al., 2012). We used the Kullback–Leibler (Cameron & Windmeijer, 1997) pseudo-*R*^2^, *R*^2^_*KL*_, to index the variance explained by the full model and semipartial-*R*^2^_*KL*_, *ΔR*^2^_*KL*_, as a measure of effect size for the main effects and interactions. We did not analyze trial time because analyses revealed no differences in performance accuracy as a function of S+ orientation or rotation plane.

### Results

#### Performance accuracy

Of the 1,152 testing trials, 1,146 (99.5%) completed trials were analyzed. In the logistic regression model, we accounted for rotation plane–fish-specific change over time by included main effects of rotation plane and fish, linear effects of trial number and session number, and all corresponding two-way, three-way, and four-way interactions. We also included main effects of S+ orientation and two-way and three-way interactions among fish, rotation plane, and S+ orientation to assess fish-specific differences across rotation plane and S+ orientation conditions. Finally, we included a main effect of S+ location and its interaction with fish to assess potential side biases, *R*^*2*^_*KL*_ = 11.61%. Type III Wald tests of interactions and main effects were used. None of the three-way and four-way interactions were statistically significant, all *p* > .30, all *R*^2^_KL_ < 0.6%. Overall model-estimated accuracy was significantly better than chance (i.e., 50%), *M* = 59.8%, *SE* = 2.1%, *Z* = 4.58, *p* < .001, 95% CI [55.6%, 63.8%].

To assess change over trials or sessions, we examined interactions involving change over sessions and change over trials by fish and rotation plane. None of the main or interaction effects involving trial number and session number were statistically significant, all *p* > .05, *ΔR*^2^_*KL*_ < 0.5%, which suggested that performance did not improve or decline significantly over time for any of the fish. Results suggested that there were no differences in performance between rotation plane. The interaction between fish and rotation plane was not statistically significant, χ^2^(3) = 0.94, *p* = .817, *ΔR*^2^_*KL*_ = 0.06%. The main effect of rotation plane was also not statistically significant, χ^2^(1) = 0.13, *p* = .721, *ΔR*^*2*^_*KL*_ = 0.01%.

**Fig. 5:**
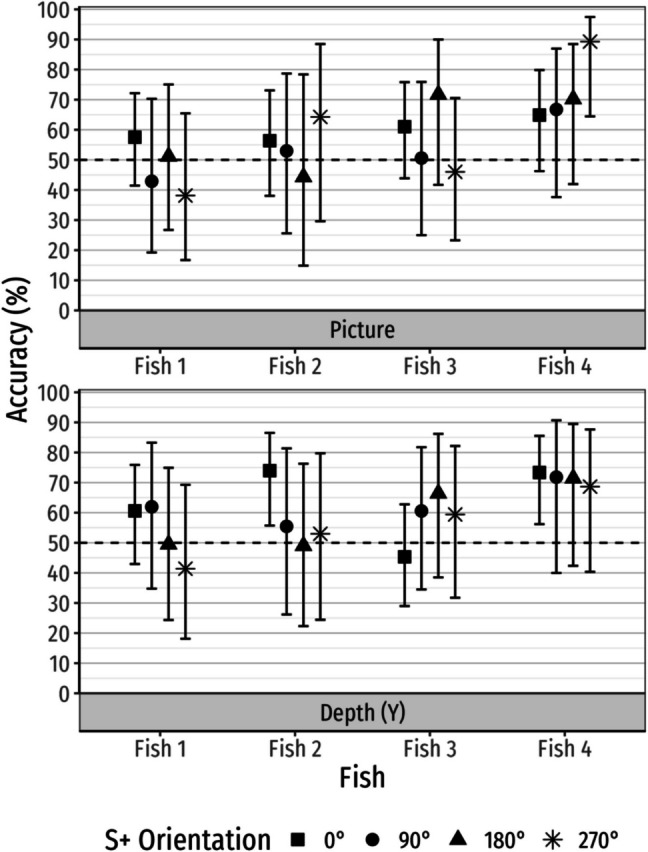
Performance accuracy of individual fish as a function of rotation plane and aspect angle in Experiment 3. Accuracies are shown along with Tukey-adjusted 95% confidence intervals

Similarly, results suggested that there were no differences in performance among S+ orientations. Neither the interaction between fish and S+ orientation, χ^2^(9) = 13.40, *p* = .145, *ΔR*^2^_*KL*_ = 0.81%, nor the main effect of S+ orientation, χ^2^(3) = 0.65, *p* = .884, *ΔR*^2^_*KL*_ = 0.04%, were statistically significant. The performance of each fish in each rotation plane across S+ orientations is shown in Fig. 5: Fish 1 and Fish 3 did not perform significantly better than chance in either rotation plane at any S+ orientation. Fish 2 only performed significantly better than chance in the Depth (Y) rotation plane at 0°, *M* = 74.0%, 95% CI [55.7%, 86.5%]. Fish 4 performed significantly better chance when the S+ was rotated 270° in the picture plane, *M* = 89.3%, 95% CI [64.5%, 97.5%] and in the Depth (Y) rotation plane at 0°, *M* = 73.4%, 95% CI [56.2%, 85.5%].

There was a statistically significant main effect of fish, χ^2^(3) = 20.49, *p* < .001, *ΔR*^2^_*KL*_ = 1.45%, suggesting that performance differed among fish (Table S5). Among the four fish, Fish 1 and Fish 2 did not perform statistically significantly better than chance, *Z* = 0.11, *p* = .910 and *Z* = 1.47, *p* = .142, respectively. Fish 3 and Fish 4, however, both performed significantly better than chance, *Z* = 2.15, *p* = .032 and *Z* = 5.91, *p* < .001, respectively. There was a statistically significant interaction between fish and S+ position, χ^2^(3) = 97.97, *p* < .001, *ΔR*^2^_*KL*_ = 7.01%, suggesting the fish were prone to side biases. S+ position-specific performance for each fish is provided in Table S6. Fish 1 and 2 performed significantly better when the S+ was on the left, whereas Fish 3 and 4 performed better when the S+ was on the right.

#### Meta-analytic comparisons of performance among experiments

**Fig. 6:**
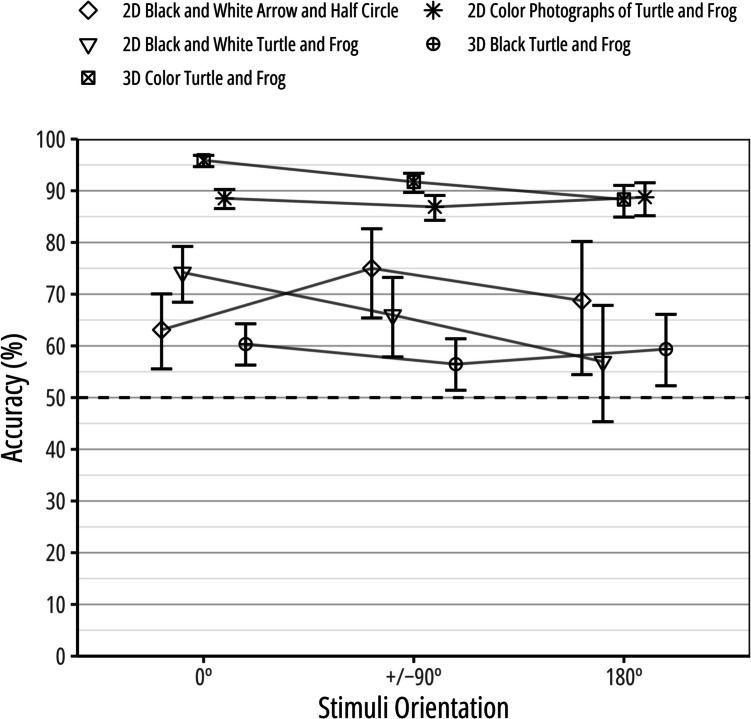
Goldfish performance accuracy across several object constancy studies*.* The 2D black and white simple stimuli (arrow and half circle) and 2D black and white complex stimuli (line drawings of turtle and frog) data are from DeLong et al. (2018), and the 2D color photographs of turtle and frog data are from Wegman et al. (2022). The 3D color turtle and frog data are from Experiment 1 and the 3D black turtle and frog data are from Experiment 3 of the current study

We compared performance between Experiments 1, 2, and 3 with a fixed-effects meta-analysis of the correct and incorrect choices in both experiments using the log-odds ratio (e.g., Fleiss & Berlin, 2009) using a Tukey adjustment for multiple comparisons. Performance was significantly better in Experiment 2 (*Accuracy* = 97.6%) than in both Experiment 1 (accuracy = 93.2%), log-odds ratio = 1.10, *SE* = 0.19,* Z* = 5.76, *p* < .001, 95% CI [0.66, 1.54], and Experiment 3 (accuracy = 59.8%), log-odds ratio = 3.36, *SE* = 0.18,* Z* = 18.30, *p* < .001, 95% CI [2.94, 3.79]. Confirming H3, performance was significantly better in Experiment 1 than in Experiment 3, log-odds ratio = 2.26, *SE* = 0.10,* Z* = 22.91, *p* < .001, 95% CI [2.04, 2.49]. Figure 6 shows performance in the current study compared with other studies (DeLong et al., 2018; Wegman et al., 2022).

A similar fixed-effects meta-analysis of Experiments 1 and 3 and data from DeLong et al. (2018) and Wegman et al. (2022) was used to compare performance (i) between 3D color turtle and frog (Experiment 1) and 2D color photographs of turtles and frogs (Wegman et al., 2022), and (ii) 3D black turtles and frogs (Experiment 3) and 2D black shapes and line drawings stimuli (DeLong et al., 2018). First, when color cues were available, performance was statistically significantly better for 3D stimuli (Experiment 1, accuracy = 93.2%) than 2D stimuli (Wegman et al., 2022; accuracy = 88.0%), log-odds ratio = 0.63, *SE* = 0.10,* Z* = 6.21, *p* < .001, 95% CI [0.36, 0.91]. Second, when color cues were removed (Experiment 3; accuracy = 58.9%), performance was statistically significantly worse than goldfish who have only viewed objects that lack color cues (DeLong et al., 2018; accuracy = 68%), log-odds ratio = −0.41, *SE* = 0.10,* Z* = −4.14, *p* < .001, 95% CI [−0.60, −0.22].

### Discussion

In Experiment 3, the fish did not display object constancy with the achromatic stimuli and showed overall poor performance (*M* = 59.8%). The fish struggled to learn to discriminate between the achromatic stimuli at 0°, requiring 342–366 trials to achieve the training criterion of 67% (compared with 100 trials or less to reach near ceiling performance with the chromatic stimuli in Experiment 1). In the test phase, only half of the fish subjects performed better than chance overall. Two fish successfully discriminated between the stimuli at 0° (training aspect), and only one of four fish performed better than chance at 270° in one rotation plane. Discriminating the achromatic stimuli appeared to be a much more difficult task for the fish, especially when the stimuli were rotated to novel aspect angles.

The goldfish in Experiment 3 performed worse than the archerfish in Newport et al.’s (2018) study, who recognized 2D faces (same skin color) rotated by 30° or 60° but not at 90°. The four goldfish in the current study always failed at 180°, unlike the Medaka fish in Wang and Takeuchi’s (2017) study that discriminated between pairs of same-colored 3D objects rotated 180°. The goldfish in the current study performed best at 0°, which is the aspect angle they were most familiar with from the training phase. Good performance at 0° matches the performance of cichlids viewing pairs of turtles and frogs at 0° that were very close in color (e.g., two dark green, two light green, or two gray stimuli; Schluessel et al., 2014).

## General Discussion

This was the first study to investigate the ability of fish to recognize the same 3D stimuli with and without color cues presented at four aspect angles in both picture and depth planes. As predicted, the goldfish were significantly more successful at recognizing the turtle and frog stimuli with color cues available (Experiments 1 and 2) than they were when the stimuli were achromatic (Experiment 3). Only two of four fish were able to discriminate between the black turtles and frogs at some aspect angles and showed overall performance above chance. When viewing rotated chromatic stimuli, fish continued to perform well regardless of rotation scheme (whether one or both stimuli were rotated). The pattern of results across all three experiments suggested a mix of both viewpoint-invariant and viewpoint-dependent processes at work.

The fish achieved object constancy in Experiments 1 and 2 with chromatic stimuli. These results are in agreement with those of the cichlids viewing 3D models of turtles and frogs of various colors (Schluessel et al., 2014). Thus, at least two species of fish, both freshwater-dwelling and possessing color vision, can succeed at this task. Goldfish have tetrachromatic color vision (Neumeyer, 1992, 2003a) and cichlids appear to have tetrachromatic or pentachromatic color vision (Sabbah et al., 2010). Color vision likely plays a central role in the visual perception and behavior of both species. Cichlid and goldfish could use coloration patterns to identify individuals and choose mates (Jordan et al., 2003; Neumeyer, 2003a, 2003b). Individual goldfish vary in coloration pattern (they can be orange, red, white, gray, or a combination of those colors). Goldfish are omnivores that forage by digging through the substrate and or browsing on plants like algae. They will eat insect larvae, eggs, invertebrates, and small crustaceans. Vision (specifically color perception) may help them identify food objects against the background of the substrate or help them to identify a landmark used to locate a food source, territory, or refuge (Douglas, 1996). Although vision is important for goldfish, they can detect odorants associated with feeding, reproduction, and aggregation (Sato & Sorenson, 2018). Object recognition is most likely multimodal in their natural habitat, but we controlled for odor and in the present study, so they were limited to visual information.

Goldfish may be like some other animal species, such as rhesus monkeys and fruit flies, where color may be learned faster than other visual object characteristics, like shape or size (e.g., Baxter & Gaffan, 2007; Benelli & Canale, 2012; Papaj & Lewis, 2012). There is evidence that guppies (who have color vision and likely utilize color cues for mate choice), learned to discriminate between the colors red and yellow faster than between two shapes (a circle and a triangle; Lucon-Xiccato et al., 2019). While the primacy of color can be an advantage for fish learning to discriminate among stimuli with color cues, it may also be a disadvantage if color cues are not present.

The results of Experiment 3, showing that the goldfish largely failed to achieve object constancy with achromatic stimuli but showed exceptional performance with chromatic stimuli raises the possibility that many fish may have been using color instead of shape to perform the discrimination. There is no need to represent the shape of an object if you can reliably detect color differences (e.g., fish with the red and yellow frog as their S+ can just choose the red and yellow stimulus and reject the green stimulus without building a representation of the shape of the objects). However, since color is important to goldfish, they may immediately pick up on that cue but also encode shape information when necessary. The prior experience of the individual subject (e.g., with objects that do or do not have color cues) and stimulus type used in the study (e.g., 3D vs. 2D) likely factor into what type of object representation fish utilize.

It is interesting that the fish (Fish 4) that showed the highest performance in Experiment 3 with the black turtles and frogs (*M* = 73%) is one of the three subjects that did not participate in Experiments 1 and 2 with the green/gray turtles and yellow/red frogs. Fish 3, who performed better than chance overall, also did not participate in the first two experiments. Fish 3 and 4 had just completed another study with two black stimuli made from LEGO® blocks (C. DeLong, unpublished data), and had never participated in any other two-alternative forced-choice task with chromatic stimuli. Perhaps Fish 3 and Fish 4 learned to pick up on cues other than color during object discrimination because they had never been able to use color cues? Of the two fish that participated in Experiments 1 and 2, one failed to make it to the test phase and one did not perform significantly better than chance in the test phase of Experiment 3. The extensive experience of the fish in the first two experiments with chromatic stimuli may have taught them to rely on color cues. Comparing across studies, goldfish who have first learned an object discrimination when stimuli have color cues perform worse on an object constancy task when color cues are removed in a follow-up experiment (Experiment 1 vs. Experiment 3, current study) than goldfish who have only viewed rotated objects that lack color cues (DeLong et al., 2018; see Figure 6).

Both the prior experience of the individual and their exposure to the types of objects used in the study likely shapes both performance on this type of task and use of cues. We deliberately chose to include subjects with and without experience discriminating among chromatic stimuli in Experiment 3. We also gave two subjects continuous 24-hour contact with the plastic turtle and frog models in Experiments 1 and 2 to see whether they would show an advantage in recognizing rotated stimuli over the other subjects. Fish 1 and 6 had access to the fish and turtle models in their home tank and could swim around them and view them from every aspect angle. This exposure to the models during training and during the test phases of Experiments 1 and 2 did not seem to confer any advantage to these two subjects. They showed no difference in accuracy or trial time from the other subjects during the training phase before Experiment 1 or during testing in Experiments 1 and 2. This could mean that a viewpoint-invariant representation could be attained without viewing all aspects of the stimulus or that the task with colored stimuli was overall so easy for the fish that they did not need additional exposure to the stimuli. Or perhaps they actually spent little time exploring the stimuli in their home tanks (we did observe them swimming around the stimuli occasionally). Unfortunately, neither of these subjects participated in Experiment 3 so it is unclear whether they would have had an advantage with the more difficult achromatic object discrimination. Future object constancy studies could more systematically explore the effect of prior experience and amount of exposure to the stimuli.

In addition to considering prior experience in interpreting the performance of the fish within and across studies (see Fig. 6), we should also consider other facets of the stimuli. The 3D color turtle and frog (Experiment 1) and 2D color photographs of turtles and frogs (Wegman et al., 2022) had surface features (texture, shading, color). Whereas the 2D black shapes and line drawings stimuli (DeLong et al., 2018) and 3D black turtles and frogs (Experiment 3) lacked color, texture, and shading. The black paint used for the turtles and frogs deemphasized any texture cues (like on the turtle shell or the back of the frog) and likely made the outer outline/edge of the stimulus the main shape cue. The goldfish clearly performed better on stimuli with surface features. Further, they performed best with 3D stimuli in this study (where the surface features were likely more clearly detected) compared with 2D color stimuli in Wegman et al. (2022). Wood and Wood (2024) found that newborn chicks could learn to recognize objects across novel viewpoints when objects contained surface features, but not when the objects were line drawings. Similarly, pigeons form different representations of stimuli if they are shaded grayscale objects with surface features or line drawings (Peissig et al., 2005). Line drawings emphasize object edges, but edges may not be enough to form the kind of robust object representation that may be required for achieving object constancy.

A limitation of the current study is the order in which the fish were tested. All fish that were exposed to both the chromatic stimuli in Experiments 1 and 2 and the achromatic stimuli in Experiment 3 received the chromatic stimuli first. That the two fish that performed best in Experiment 3 did not participate in Experiment 1 (and had no experience with full color stimuli) suggests that future studies on this topic should assign half the subjects to the achromatic object discrimination first and the other half to the chromatic object discrimination stimuli first. Similarly, all fish were tested with the matched rotation scheme in Experiment 1 before they were tested with the nonmatched rotation scheme in Experiment 2. Counterbalancing would be ideal in future research. It would be beneficial to replicate this study, particularly Experiment 3, before drawing firm conclusions about the ability of fish to recognize rotated achromatic objects.

Another limitation of the current study is that we had only five to eight subjects per experiment. This is similar to other studies in which there were two to five fish per experiment (Frech et al., 2012; Gierszewski et al., 2013; Neumeyer, 1992, 2003b; Newport et al., 2016; Wyzisk & Neumeyer, 2007). We should interpret our results cautiously given that we had few subjects, and individual differences were apparent. However, we collected many trials for each of our subjects (e.g., each fish had 432 test trials in Experiment 1) to compensate for having fewer subjects. The recent trend for a big-team science approach to collecting data across many laboratories (e.g., ManyX groups such as ManyBirds, Lambert et al., 2022; ManyDogs Project et al., 2023; ManyPrimates et al., 2019) is a good way to greatly increase sample sizes, and soon some types of studies will have more fish subjects. But studies like the current one that require longer investments of training and testing time may still have limited sample sizes.

Studies of object constancy in fish have been limited to a few species—for example, archerfish, cichlids, goldfish, and medaka—of the approximately 30,000 extant species. In order to make sense of the mixed results, we find in these studies, it would be advantageous to systematically test fish with different habitats and life histories. The development of object constancy and its expression likely is related to the ecological demands placed on the species. Fish with color vision likely show optimized performance on stimuli closely related to multicolored objects that surround them in their natural environment. Fish that forage by hitting moving prey items with a jet of water (archerfish) need to be able to identify quickly rotating objects. Fish that use vision to recognize conspecifics would have an advantage if they could spot them quickly regardless of orientation (with the exception being the face inversion effect—Wang & Takeuchi, 2017). All fish that swim in a three-dimensional world typically viewing objects from many viewpoints would benefit, at least to some degree, from object constancy, but some species may rely on it more heavily than others.

## Supplementary Information

Below is the link to the electronic supplementary material.

Supplementary file1 (PDF 254 KB)

## Data Availability

The data presented in this study are openly available in the Open Science Framework (OSF) (https://osf.io/4nhg6/).

## Code availability

The code used to analyze the data in this study are openly available in the Open Science Framework (OSF) (https://osf.io/4nhg6/).
