# Supplementary material for: Visual perception of rotated chromatic and achromatic 3D stimuli in goldfish (Carassius auratus)
Source: Learn Behav. 2025 Nov 12;54(1):37–59. doi: 10.3758/s13420-025-00687-y (PMC12999622; doi:10.3758/s13420-025-00687-y)
Supplement: Supplementary file 1 — Supplementary file1 (PDF 254 KB) [file 13420_2025_687_MOESM1_ESM.pdf]

Supplementary Material

Supplementary Tables

Table S1

Performance (%) and Trial Time (s) of Fish in Each Rotation Plane in The Test Phases of Experiments 1 and 2.

		Performance (%)			Trial Time (s)		
Fish	Rotation Plane	*M* (*SE*)	*95% CI*	Group	*M* (*SE*)	*95% CI*	Group
1	Picture	98.8 (0.9)	[91.1, 99.9]	1.1A	3.4 (0.2)	[3.0, 4.2]	1.1A
	Depth (Y)	98.2 (1.2)	[89.6, 99.7]	1.1A	4.4 (0.3)	[3.7, 5.6]	1.1AB
	Depth (X)	96.8 (1.8)	[86.1, 99.3]	1.1A	5.0 (0.3)	[4.2, 6.3]	1.1B
	Only S+ in Picture	98.9 (0.6)	[95.7, 99.7]		6.0 (0.4)	[5.4, 7.0]	1.2A
	Only S- in Picture	97.6 (1.2)	[92.1, 99.3]		5.5 (0.4)	[4.9, 6.4]	1.2A
2	Picture	98.4 (1.3)	[85.7, 99.8]	2.1B	5.1 (0.4)	[4.3, 6.5]	2.1A
	Depth (Y)	81.5 (4.2)	[67.0, 90.5]	2.1A	6.0 (0.4)	[5.0, 7.9]	2.1AB
	Depth (X)	89.6 (3.1)	[77.4, 95.6]	2.1AB	7.6 (0.6)	[6.3, 10.4]	2.1B
	Only S+ in Picture						
	Only S- in Picture						
3	Picture	98.8 (0.7)	[93.7, 99.8]	3.1B	2.5 (0.1)	[2.2, 2.9]	3.1A
	Depth (Y)	91.6 (2.8)	[79.8, 96.8]	3.1A	2.9 (0.2)	[2.5, 3.5]	3.1A
	Depth (X)	93.2 (2.6)	[81.3, 97.7]	3.1A	3.0 (0.2)	[2.6, 3.6]	3.1A
	Only S+ in Picture	98.2 (1.0)	[93.5, 99.5]		2.8 (0.1)	[2.5, 3.0]	3.2B
	Only S- in Picture	95.9 (1.8)	[88.4, 98.6]		2.4 (0.1)	[2.2, 2.7]	3.2A
4	Picture	98.1 (0.1)	[86.1, 99.8]	4.1A	3.2 (0.2)	[2.8, 3.9]	4.1A
	Depth (Y)	99.2 (0.9)	[83.9, 100.0]	4.1A	5.3 (0.4)	[4.4, 6.9]	4.1B
	Depth (X)	100.0 (0.04)	[100.0, 100.0]	4.1A	4.9 (0.3)	[4.2, 6.2]	4.1B
	Only S+ in Picture	99.3 (0.4)	[96.8, 99.9]		4.0 (0.2)	[3.7, 4.5]	4.2A
	Only S- in Picture	98.5 (0.9)	[94.1, 99.6]		6.9 (0.5)	[6.1, 8.3]	4.2B
5	Picture	97.4 (1.5)	[87.5, 99.5]	5.1A	4.2 (0.3)	[3.6, 5.3]	5.1A
	Depth (Y)	97.2 (1.4)	[88.9, 99.3]	5.1A	4.6 (0.3)	[3.9, 5.9]	5.1A
	Depth (X)	93.5 (2.4)	[82.7, 97.8]	5.1A	4.5 (0.3)	[3.8, 5.6]	5.1A
	Only S+ in Picture	99.8 (0.2)	[97.8, 100.0]		4.2 (0.2)	[3.8, 4.8]	5.2A
	Only S- in Picture	99.6 (0.4)	[95.4, 100.0]		4.4 (0.3)	[3.9, 5.1]	5.2A
6	Picture	91.4 (2.8)	[79.9, 96.6]	6.1A	3.7 (0.2)	[3.2, 4.6]	6.1A
	Depth (Y)	98.8 (1.1)	[84.7, 99.9]	6.1A	3.8 (0.2)	[3.3, 4.6]	6.1A
	Depth (X)	98.1 (1.2)	[89.6, 99.7]	6.1A	4.4 (0.3)	[3.8, 5.5]	6.1A
	Only S+ in Picture	99.8 (0.2)	[98.1, 100.0]		5.5 (0.3)	[4.9, 6.3]	6.2A
	Only S- in Picture	99.5 (0.4)	[96.3, 99.9]		4.9 (0.3)	[4.4, 5.6]	6.2A

Note. Discriminative accuracy and average trial times are given with corresponding Scheffé-adjusted 95% confidence intervals for Experiment 1 (Picture, Depth (Y-axis), and Depth (X-axis) planes). For Experiment 1, planes within a given fish that share a group did not differ significantly (e.g., accuracy did not differ significantly for Fish 2 between Plane 1 vs. Plane 3, nor between Plane 2 vs. 3, but Fish 2 performed significantly worse in Plane 2 than in Plane 1). Because there were no significant differences in performance among fish nor among planes in Experiment 2 for discriminative accuracy, differences in performance between planes for each fish were not tested, confidence intervals were not adjusted, and pairwise comparisons are not

reported in the Accuracy Group column. Average trial times are given with unadjusted 95% confidence intervals for Experiment 2.

Table S2

Performance Accuracy and Trial Time of Fish by S+ Orientation in Experiment 1 Testing

		Performance (%)			Trial Time (s)	
Fish	S+ Orientation	*M* (*SE*)	*95% CI*	Group	*M* (*SE*)	*95% CI*
1	0°	99.4 (0.5)	[97.4, 99.8]	1A	4.0 (0.2)	[3.8, 4.4]
	90°	98.2 (1.4)	[92.2, 99.6]	1A	4.0 (0.2)	[3.7, 4.5]
	180°	95.0 (2.9)	[85.2, 98.4]	1A	4.4 (0.2)	[4.0, 4.8]
	270°	97.8 (1.7)	[90.7, 99.5]	1A	4.1 (0.2)	[3.7, 4.5]
2	0°	98.4 (0.8)	[95.5, 99.5]	2B	5.7 (0.3)	[5.3, 6.4]
	90°	93.3 (3.4)	[82.8, 97.6]	2AB	5.8 (0.4)	[5.1, 6.8]
	180°	86.3 (5.4)	[72.0, 93.9]	2A	6.8 (0.6)	[5.9, 8.2]
	270°	84.2 (6.1)	[68.5, 92.9]	2A	5.8 (0.4)	[5.2, 6.8]
3	0°	93.4 (2.1)	[88.0, 96.5]	3A	2.8 (0.1)	[2.6, 2.9]
	90°	96.8 (2.0)	[89.5, 99.1]	3A	2.8 (0.1)	[2.6, 3.0]
	180°	96.9 (1.8)	[90.7, 99.0]	3A	2.8 (0.1)	[2.7, 3.1]
	270°	95.7 (2.5)	[87.1, 98.6]	3A	2.8 (0.1)	[2.6, 3.0]
4	0°	100.0 (0.1)	[99.1, 100.0]	4A	4.1 (0.2)	[3.8, 4.4]
	90°	99.8 (0.3)	[95.8, 100.0]	4A	4.1 (0.2)	[3.8, 4.5]
	180°	99.4 (0.7)	[92.7, 100.0]	4A	4.4 (0.2)	[4.0, 4.9]
	270°	99.2 (1.1)	[90.0, 99.9]	4A	4.1 (0.2)	[3.8, 4.5]
5	0°	99.6 (0.3)	[98.2, 99.9]	5B	4.3 (0.2)	[4.0, 4.7]
	90°	88.6 (4.9)	[75.4, 95.2]	5A	4.4 (0.2)	[4.0, 4.8]
	180°	91.8 (3.7)	[81.0, 96.8]	5A	4.8 (0.2)	[4.3, 5.3]
	270°	95.7 (2.8)	[85.3, 98.8]	5AB	4.4 (0.2)	[4.0, 4.9]
6	0°	99.4 (0.4)	[97.9, 99.9]	6B	3.9 (0.1)	[3.6, 4.2]
	90°	97.0 (2.1)	[88.8, 99.2]	6AB	3.9 (0.2)	[3.6, 4.3]
	180°	93.0 (3.4)	[82.8, 97.4]	6A	4.2 (0.2)	[3.8, 4.6]
	270°	95.4 (2.8)	[85.6, 98.6]	6AB	3.9 (0.2)	[3.6, 4.3]

Note. Discriminative accuracy and average trial time (in seconds) are given for Experiment 1. For Experiment 1, S+ orientations where accuracy for a given fish did not differ significantly share a group (e.g., accuracy did not differ significantly for Fish 2 between 90°, 180°, and 270°, but accuracy was significantly higher at 0° than at 180° and 270°); corresponding Tukey-adjusted 95% confidence intervals are shown. Grouping of significant and non-significant differences in average trial times is not included because there were no significant differences in trial times among S+ orientations and confidence intervals are not adjusted.

Table S3

Performance Accuracy and Trial Time in Each Rotation Plane and S+ Orientation in Experiments 1 and 2

		Performance (%)			Trial Time (s)	
Rotation Plane	S+ Orientation	*M* (*SE*)	*95% CI*	Group	*M* (*SE*)	*95% CI*
Exp 1: Picture	0°	96.3 (1.0)	[93.8, 97.8]	1A	3.4 (0.1)	[3.2, 3.6]
	90°	97.6 (1.2)	[93.8, 99.0]	1A	3.4 (0.1)	[3.2, 3.6]
	180°	96.6 (1.4)	[92.5, 98.5]	1A	3.6 (0.1)	[3.4, 3.8]
	270°	96.0 (1.6)	[91.2, 98.2]	1A	3.4 (0.1)	[3.4, 3.6]
Exp 1: Depth (Y)	0°	99.0 (0.4)	[97.9, 99.6]	2B	4.0 (0.1)	[3.8, 4.3]
	90°	88.3 (3.1)	[80.8, 93.2]	2A	4.1 (0.2)	[3.8, 4.4]
	180°	85.1 (3.4)	[77.2, 90.7]	2A	4.4 (0.2)	[4.1, 4.8]
	270°	94.2 (2.1)	[88.4, 97.2]	2A	4.1 (0.2)	[3.8, 4.4]
Exp 1: Depth (X)	0°	98.4 (0.6)	[96.8, 99.2]	3B	4.3 (0.1)	[4.1, 4.6]
	90°	95.6 (1.7)	[90.8, 98.0]	3AB	4.3 (0.2)	[4.0, 4.7]
	180°	89.2 (2.8)	[82.4, 93.6]	3A	4.7 (0.2)	[4.3, 5.1]
	270°	89.1 (3.0)	[81.8, 93.7]	3A	4.3 (0.2)	[4.0, 4.7]
Exp 2: Only S+ in Picture	0°	99.2 (0.4)	[98.0, 99.7]		4.2 (0.1)	[3.8, 4.7]
	90°	99.2 (0.5)	[96.9, 99.8]		3.7 (0.2)	[3.2, 4.5]
	180°	99.1 (0.6)	[96.8, 99.8]		3.9 (0.2)	[3.4, 4.8]
	270°	99.8 (0.2)	[98.0, 100.0]		4.4 (0.2)	[3.4, 4.8]
Exp 2: Only S- in Picture	0°	98.3 (0.7)	[96.2, 99.2]		3.8 (0.1)	[3.5, 4.3]
	90°	98.3 (1.1)	[94.0, 99.5]		4.3(0.2)	[3.6, 5.4]
	180°	98.0 (1.2)	[93.8, 99.4]		3.8 (0.2)	[3.2, 4.6]
	270°	99.6 (0.5)	[95.7, 100.0]		4.0 (0.2)	[3.5, 5.0]

Note. Discriminative accuracy is given with corresponding Tukey-adjusted 95% confidence intervals for Experiment 1 (Picture, Depth (Y), and Depth (X) planes) and average trial time (seconds) is given with unadjusted 95% confidence intervals. For Experiment 1, S+ orientations within a given rotation plane that share a group indicate that accuracy did not differ significantly (e.g., accuracy did not differ significantly in the Depth(Y) plane between 90°, 180°, and 270°, but accuracy was significantly higher at 0° than at the other orientations). Grouping of significant and non-significant differences in average trial times is not included for Experiment 1 because there were no significant differences in trial times among S+ orientations. Because there were no significant differences in accuracy among test block nor among S+ orientations in Experiment 2 (Only S+ in Picture Plane and Only S- in Picture Plane), differences in accuracy between S+ orientations for each test block are not tested, confidence intervals are not adjusted, and pairwise comparisons are not reported in the Accuracy Group column. In Experiment 2, average trial times and Scheffé-adjusted 95% confidence intervals are provided, but a Group column is not provided because none of the adjusted pairwise comparisons of trial times were statistically significant.

Table S4

Performance Accuracy and Trial Time of Fish by S+ Position in Experiments 1 and 2

			Performance (%)			Trial Time (s)	
Fish	Experiment	S+ Position	*M* (*SE*)	*95% CI (%)*	Group	*M* (*SE*)	*95% CI*
1	1	Left	99.4 (0.5)	[96.7, 99.9]	1B	4.2 (0.2)	[3.9, 4.5]
		Right	94.5 (1.9)	[89.3, 97.2]	1A	4.1 (0.2)	[3.8, 4.4]
	2	Left	98.2 (0.1)	[95.4, 99.3]			
		Right	97.8 (0.1)	[94.8, 99.1]			
2	1	Left	94.4 (2.1)	[88.4, 97.4]	2A	6.1 (0.3)	[5.6, 6.9]
		Right	91.1 (2.9)	[83.5, 95.4]	2A	5.9 (0.3)	[5.4, 6.6]
	2	Left					
		Right					
3	1	Left	87.0 (3.3)	[79.1, 92.3]	3A	2.8 (0.1)	[2.6, 3.0]
		Right	98.8 (0.6)	[96.6, 99.6]	3B	2.8 (0.1)	[2.6, 3.0]
	2	Left	97.0 (1.3)	[93.0, 98.7]			
		Right	96.3 (1.4)	[92.4, 98.3]			
4	1	Left	99.3 (0.9)	[93.2, 99.9]	4A	4.2 (0.2)	[3.9, 4.6]
		Right	99.9 (0.2)	[98.1, 100.0]	4A	4.1 (0.2)	[3.8, 4.5]
	2	Left	98.9 (0.6)	[96.7, 99.6]			
		Right	98.6 (0.7)	[96.2, 99.5]			
5	1	Left	98.0 (1.0)	[94.8, 99.3]	5B	4.5 (0.2)	[4.2, 4.9]
		Right	93.5 (2.0)	[88.1, 96.5]	5A	4.4 (0.2)	[4.1, 4.8]
	2	Left	99.7 (0.3)	[97.9, 100.0]			
		Right	99.7 (0.3)	[97.6, 100.0]			
6	1	Left	96.6 (1.6)	[91.8, 98.7]	6A	4.0 (0.2)	[3.7, 4.3]
		Right	97.8 (1.1)	[94.0, 99.2]	6A	3.9 (0.1)	[3.6, 4.2]
	2	Left	99.6 (0.3)	[98.1, 99.9]			
		Right	99.5 (0.4)	[97.8, 99.9]			

Note. Discriminative accuracy and average trial time are given. For Experiment 1, S+ positions within a given fish that share a group indicate accuracy did not differ significantly (e.g., accuracy did not differ significantly for Fish 2 when the S+ was on the Left vs. Right, but Fish 1 performed significantly better when the S+ was on the Left than on the Right); Tukey-adjusted confidence intervals are provided. Because we could not test for significant differences in accuracy between S+ positions by fish in Experiment 2, differences in accuracy between S+ positions for each fish are not tested, confidence intervals are not adjusted, and pairwise comparisons are not reported in the Accuracy Group column. Grouping of significant and non-significant differences in average trial times is not included because there we could not test for differences in trial time between S+ positions by fish and confidence intervals are not adjusted.

Table S5

Performance Accuracy of Fish by S+ Assignment in Experiment 3

Fish	S+ Assigned	*M*	*95% CI (%)*
1	Turtle	50.4	[43.3, 57.5]
2	Frog	56.5	[47.8, 64.7]
3	Turtle	57.9	[50.7, 64.8]
4	Frog	73.0	[66.0, 78.9]

Note. Fish with a frog S+ (Fish 2 and 4; $M = 65.2\%$, 95% CI: [59.4%, 70.5%]) performed significantly better than fish with a turtle S+ (Fish 1 and 3; $M = 54.2\%$, 95% CI: [49.1%, 59.2%]), $OR = 1.58$, $SE = 0.24$, 95% CI: [1.17, 2.13], $Z = 3.02$, $p < .001$. For the two fish with a frog S+, Fish 4 performed significantly better than Fish 2, $OR = 2.08$, $SE = 0.49$, 95% CI: [1.31, 3.31], $Z = 3.09$, $p = .004$. For the two fish with a turtle S+, the performance of Fish 1 and Fish 3 did not differ significantly, $OR = 1.35$, $SE = 0.28$, 95% CI: [0.90, 2.03], $Z = 1.46$, $p = .269$.

Table S6

Performance Accuracy of Fish by S+ Position in Experiment 3

		Performance (%)			
Fish	S+ Position	*M* (*SE*)	*95% CI (%)*	*Z*	*p*
1	Left	57.1 (4.6)	[47.9, 65.8]	2.14	.032
	Right	43.7 (4.8)	[34.6, 53.3]		
2	Left	84.0 (3.4)	[76.1, 89.6]	8.82	< .001
	Right	24.3 (4.0)	[17.2, 33.1]		
3	Left	51.5 (4.8)	[42.2, 60.6]	-2.06	.040
	Right	64.1 (4.6)	[54.7, 72.5]		
4	Left	64.3 (4.7)	[54.6, 73.0]	-2.86	.004
	Right	80.1 (3.7)	[71.9, 86.4]		

Note. *Z*-statistics and *p*-values for post-hoc tests comparing performance between S+ positions (Left vs. Right) are provided.

Supplementary Figures

Figure S1

Average Trial Times for Individual Fish in Each Rotation Plane in Experiment 1

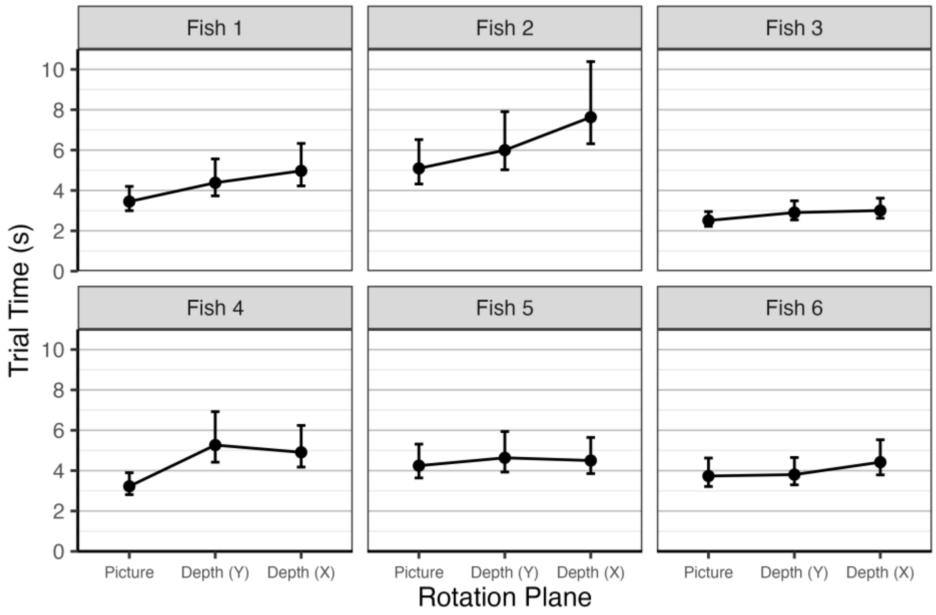

Note. Average trial times are shown along with 95% Scheffé-adjusted simultaneous confidence intervals. Overlapping confidence intervals do not necessarily imply non-significant differences between rotation planes.
